# Bacteriocin-like peptides encoded by a horizontally acquired island mediate *Neisseria gonorrhoeae* autolysis

**DOI:** 10.1371/journal.pbio.3003001

**Published:** 2025-02-05

**Authors:** Katy Poncin, Samantha A. McKeand, Hayley Lavender, Kacper Kurzyp, Odile B. Harrison, Annabell Roberti, Charlotte Melia, Errin Johnson, Martin C. J. Maiden, David R. Greaves, Rachel Exley, Christoph M. Tang

**Affiliations:** 1 Sir William Dunn School of Pathology, South Parks Road, University of Oxford, Oxford, United Kingdom; 2 Infectious Disease Epidemiology Unit, Nuffield Department of Population Health, Old Road Campus, University of Oxford, Oxford, United Kingdom; 3 Department of Biology, South Parks Road, University of Oxford, Oxford, United Kingdom; University of California Davis School of Medicine, UNITED STATES OF AMERICA

## Abstract

*Neisseria gonorrhoeae* is a human-specific pathogen that causes the important sexually transmitted infection, gonorrhoea, an inflammatory condition of the genitourinary tract. The bacterium is closely related to the meningococcus, a leading cause of bacterial meningitis. Both these invasive bacterial species undergo autolysis when in the stationary phase of growth. Autolysis is a form of programmed cell death (PCD) which is part of the life cycle of remarkably few bacteria and poses an evolutionary conundrum as altruistic death provides no obvious benefit for single-celled organisms. Here, we searched for genes present in these 2 invasive species but not in other members of the *Neisseria* genus. We identified a ~3.4 kb horizontally acquired region, we termed the *nap* island, which is largely restricted to the gonococcus and meningococcus. The *nap* island in the gonococcus encodes 3 cationic, bacteriocin-like peptides which have no detectable antimicrobial activity. Instead, the gonococcal *N**eisseria* autolysis peptides (Naps) promote autolytic cell death when bacteria enter the stationary phase of growth. Furthermore, strains lacking the Naps exhibit reduced autolysis in assays of PCD. Expression of Naps is likely to be phase variable, explaining how PCD could have arisen in these important human pathogens. NapC also induces lysis of human cells, so the peptides are likely to have multiple roles during colonisation and disease. The acquisition of the nap island contributed to the emergence of PCD in the gonococcus and meningococcus and potentially to the appearance of invasive disease in *Neisseria* spp.

## Introduction

*Neisseria gonorrhoeae*, the gonococcus, causes the sexually transmitted disease, gonorrhoea, a major worldwide public health concern. This human-specific pathogen colonises the mucosal surfaces of the urogenital tract and other sites where it elicits a pronounced inflammatory response [1]. Both *N*. *gonorrhoeae* and the closely related pathogen *Neisseria meningitidis* undergo autolysis, a form of programmed cell death (PCD), as part of their life cycle during the stationary phase of growth [2]. Although autolysis in *Neisseria* was described over a century ago [3], little is known about the pathways leading to PCD in these species or indeed in any gram-negative bacterium.

Gonococcal autolysis begins with remodelling of peptidoglycan through the activity of enzymes including the lytic transglycosylase, LtgA [4], and subsequent loss of integrity of the outer membrane, through unknown mechanisms, leads to cell death [5]. In contrast, the mechanisms underlying autolysis of *Streptococcus pneumoniae* are well understood. In this gram-positive invasive pathogen that inhabits the mucosal surface of the upper respiratory tract, activation of pneumococcal LytA, a cell wall-bound amidase, leads to degradation of the peptidoglycan cell wall at teichoic acid-rich areas [6]. Autolysis prevents phagocytosis of *S*. *pneumoniae* and liberates the cytotoxin pneumolysin and PAMPs which trigger host cell death and/or responses [7]. Higher rates of PCD in the pneumococcus correlate with the propensity of pneumococcal strains to cause invasive disease [8]. Therefore, PCD could benefit the pneumococcus *in vivo* by promoting local tissue damage and release of nutrients; however, how suicide evolves in single-celled organisms presents an evolutionary conundrum [9]. Computational studies suggest that PCD in unicellular organisms can only be an adaptive trait if it is a stochastic event [10,11], as in *S*. *pneumoniae* where *lytA* expression is subject to ON:OFF switching through phase variation [12].

To identify genes which associated with invasive potential, we performed comparative genomic analyses of >24,700 *Neisseria* genomes on PubMLST (https://pubmlst.org/) to genetic elements which are present in the invasive species, *N*. *gonorrhoeae* and *N*. *meningitidis*, but absent from the other members of the genus. This search revealed a ~3.4 kb horizontally acquired island which we designated the *nap* island. The *nap* island is found in *N*. *gonorrhoeae*, *N*. *meningitidis*, and only a few isolates of the noninvasive species *Neisseria bergeri* and *Neisseria lactamica*. In the gonococcus, this region encodes 4 peptides; 3 peptides, NapA, NapB, and NapC are cationic, with features of class II microcins, i.e., size <10 kDa with a double glycine (GG) motif [13]; the GG motif is a signal for cleavage by C39 peptidases prior to secretion [14,15]. The *nap* island also harbours genes encoding for a putative regulator (NapR), a C39 peptidase (NapP), and an export channel (NapF) related to FapF in *Pseudomonas aeruginosa* [16]. We show that, distinct from class II microcins secreted by gram-positive bacteria, the gonococcal peptides do not mediate killing of competitor bacterial species. Instead, the *N*. *gonorrhoeae* autolysis peptides (Naps) specifically promote PCD of bacteria specifically in the stationary phase of growth but no other stages in their life cycle. Furthermore, mutants lacking Naps display reduced death during stationary phase and reduced autolysis following nutrient deprivation. NapC is also cytotoxic to red blood cells (RBCs), so the acquisition of the *nap* island may help bacteria shape the local environment at mucosal surfaces in vivo. Genomic analyses suggest that NapR and NapC are phase variable, due to the presence of homo-polymeric nucleotide tracts of different lengths in their open reading frames. This would lead to the stochastic appearance within a clonal population of 2 populations of bacteria, “altruistic” autolytic bacteria and their sibling beneficiaries, indicating that PCD might have evolved in the gonococcus through kin selection. Autolysis would liberate cell contents including nutrients and DNA from bacteria that could be utilised by siblings for growth and genetic diversification, respectively. The pneumococcus, which is a naturally transformable diplococcus, similar to *N*. *gonorrhoeae* and *N*. *meningitidis*, also displays phase variable PCD [12]. Thus, phase variable PCD may operate at an exquisitely local level with adjacent, non-autolytic, first-degree relatives, i.e., nearest and dearest, the main beneficiaries from dead/dying cells. The secretion of cytotoxic NapC and the release of PAMPs following cell lysis indicate that the acquisition of the *nap* island and PCD may have been an important step in the emergence of the invasive phenotype in *Neisseria* spp.

## Results

### Identification and conservation of the nap island

**Fig 1:**
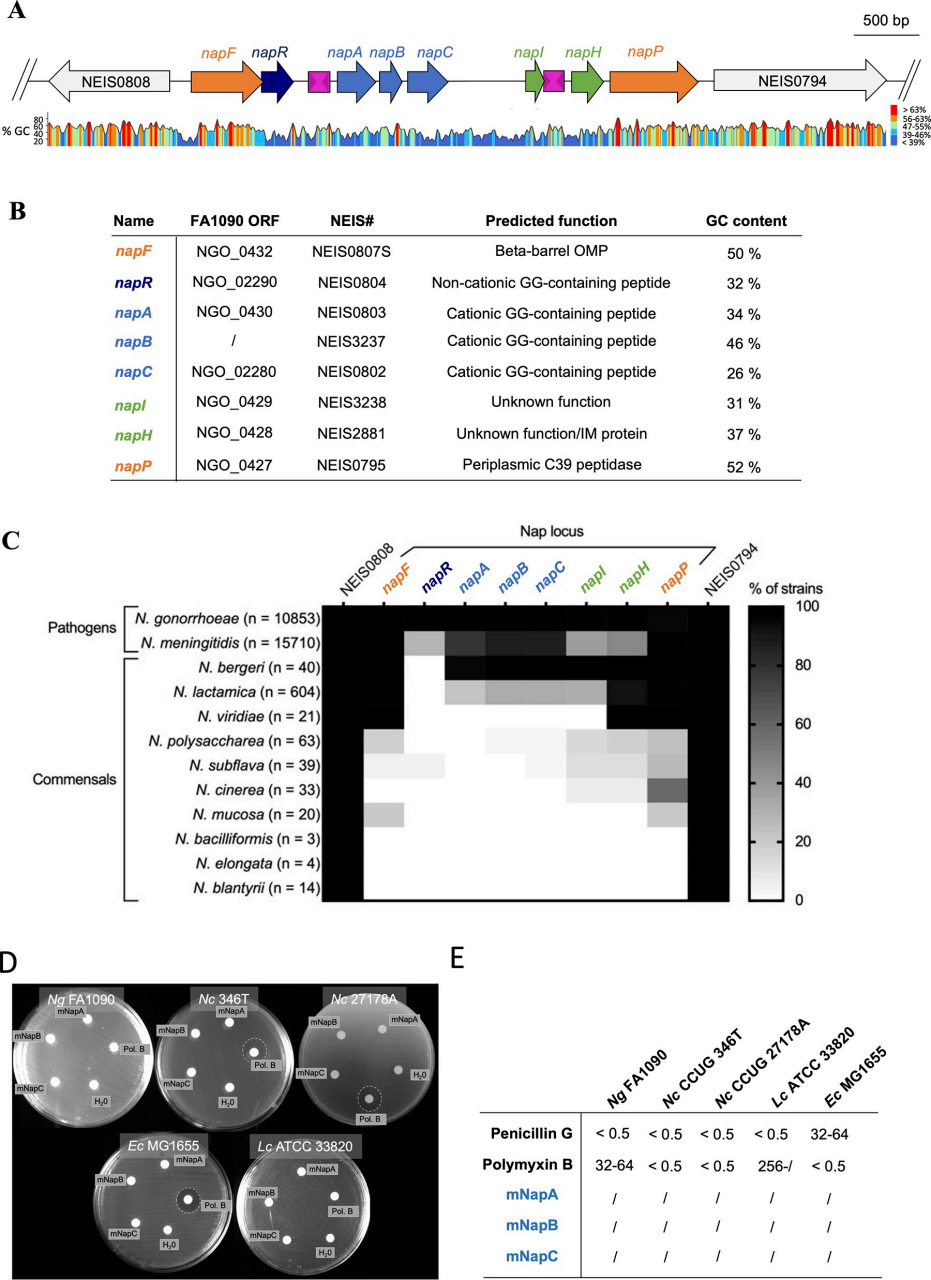
The *nap* island organisation and the absence of mNaps antimicrobial activity. **(A)** Organisation of the *nap* island in *Neisseria gonorrhoeae* FA1090. Grey arrows, flanking genes (NEIS0808, pseudouridine synthase; NEIS0794, histidyl-tRNA synthase); blue arrows, genes coding for GG-containing peptides; orange arrows, genes predicted to be involved in peptide processing and secretion; green arrow, gene of unknown function; pink boxes, Correia elements. Percentage of GC content was plotted with Snap Gene Viewer. **(B)** Table detailing predicted function of genes in the *nap* locus. Details are available in S1 Fig. **(C)** Conservation of the *nap* island and flanking genes among *Neisseria* spp. Colours are scaled in percent of strain representatives possessing a homologue within each species. The data underlying this figure can be found in S2 Table. **(D)** mNaps (50 μm) were added to disks which were then placed on lawns of *N*. *gonorrhoeae* FA1090 (*Ng*), *N*. *cinerea* (*Nc*) CCUG 346T or 27178A, *L*. *crispatus* (*Lc*) ATCC 33820, and *E*. *coli* (*Ec*) MG1655 on solid media. Water and polymyxin B (50 μm) were used as negative and positive controls, respectively. Plates were incubated for 24 h and zones of growth inhibition (dotted lines) recorded. No growth inhibition was observed for any mNap (*n* = 3). Source data is in the file S1 Data. **(E)** MICs were determined by incubating bacteria in rich medium for 24 h with antibiotics (controls) or mNaps, starting from 256 μg/ml to 0.5 μg/ml in 2-fold dilutions. Bacteria were then spotted onto agar plates, which were incubated for 24 h. MBCs were the lowest concentration at which no growth was visible on plates. The mNaps did not inhibit the growth of any strain (shown by /, *n* = 3). The data underlying this figure can be found in S1 and S2 Data files.

We searched for pathogen-specific genes in *Neisseria* spp. to further understand the basis of the invasive phenotype of some species in this genus that usually colonises mucosal surfaces asymptomatically. Using MaGe [17], we identified a ~3.4 kb region in the gonococcal genome, which we designated the *nap* island (**Figs 1A** and S**1**). This region is found in *N*. *gonorrhoeae*, with a related locus present in *N*. *meningitidis*. However, the *nap* island is largely absent from noninvasive *Neisseria* spp. (S**2 Fig**). The GC content of the *nap* island (38%) is significantly lower than rest of the genome (53%, **Fig 1A**), indicating that it was likely acquired by horizontal gene transfer (HGT) from an extant source. Consistent with this, *napC*, a gene in the centre of the *nap* island, encodes a protein with 68.9% of identity with a protein from *Inoviridae* phages (S**1 Fig**); these phages are associated with *Neisseria* spp. as 57 *Inoviridae* sequences are found in *Neisseria* CRISPR spacers (GenBank accession number DAS81996.1). One end of the island is flanked by *napP*, encoding a potential C39 peptidase predicted to process GG-containing peptides prior to secretion [14]. At the other end of the island, *napF* encodes a homologue of FapF, an outer membrane protein in *Pseudomonas aeruginosa* involved in the secretion of amyloidogenic peptides processed by a C39 peptidase [16]. Of note, both *napP* and *napF* have a different GC content compared with the rest of the *nap* island (respectively, 52% and 50%), suggesting that these 2 genes might have a different origin from the central portion of the island.

The gonococcal *nap* island contains 3 genes (*napABC*) coding for predicted α-helical cationic peptides with GG-motifs **(Fig 1B)**. After cleavage at the GG sequence, mature mNapA, mNapB, and mNapC are predicted to contain 36, 16, and 31 amino acids, with pIs of 9, 10, and 10.1, respectively (S**1 Fig**). Downstream of *napABC* (**Figs 1A** and **S1**) is *napI* which shares 30% of amino acid identity with a domain in LagC, the immunity protein for the bacteriocin, lactococcin G [18] (**S1 Fig**). *napR* is upstream of *napABC* and encodes a peptide with a putative DNA-binding domain (S**1 Fig**) so is a potential regulator of the island. Finally, *napH* is predicted to encode an inner membrane protein with a high methionine content with 3 transmembrane α-helices seen in some bacteriophage pore-forming holins [19] (S**1 Fig**).

Of note, *napR* contains a poly-G tract of 6 to 12 residues in its 5′ region, indicating that it is likely to be phase variable (S**1 Fig** and **S1 Table**). In *N*. *gonorrhoeae* FA1090, *napR* has 7 guanines, the most common allele (in 6,548 out of 10,854 isolates), leading to loss of the GG-leader sequence, so the peptide is predicted to be cytoplasmic. In addition, *napC* is also probably phase-variable, as it contains a poly-T tract towards its 5′ end resulting typically in a cationic (9xT) or non-cationic (8xT) peptide (S**1 Fig** and **S1 Table**).

The distribution of the *nap* island within *Neisseria* spp. was examined in more detail using PubMLST [20]. Whole genome sequences of >24,700 *Neisseria* spp. isolates were inspected, with results confirming that most commensal species lack the *nap* island with 2 exceptions (**Fig 1C**). The *nap* island is found in a proportion of isolates of *Neisseria bergeri* (38/40 have *napA*) and *Neisseria lactamica* (138/604 have *napA*), but no other noninvasive species (**Figs 1C and** S**2** and **S2 Table** for details).

### Cationic Naps do not inhibit the growth of competitor bacteria

Most class II microcins are antibacterial [21]. To determine whether the cationic Naps possess antibacterial activity, mature versions of the peptides, mNapA, mNapB, and mNapC, were synthesised and their ability to kill competitor bacteria assessed. First, we performed disc diffusion assays against *N*. *gonorrhoeae*, *Neisseria cinerea*, *Escherichia coli*, and the gram-positive bacterium *Lactobacillus crispatus*, an inhabitant of the genital tract [22]. As microcins are bactericidal in the nanomolar range [23], assays were performed using discs with 50 μm of each mNap, and with polymyxin B as a positive control. As expected [24], growth of *N*. *cinerea* and *E*. *coli* was inhibited around discs containing polymyxin B, while there was no clearance of *N*. *gonorrhoeae* or *L*. *crispatus* around these discs (**Fig 1D**). In contrast, none of the mNaps inhibited the growth of any strain. We also measured the minimal bactericidal concentration (MBC) of each mNap by broth dilution against the bacteria. Bacteria were incubated with micromolar concentrations of mNaps or polymyxin B for 24 h. Of note, the mNaps failed to inhibit bacterial growth even at the highest concentration (256 μm, **Fig 1E**).

As some bactericidal mechanisms are contact-dependent [25], we performed co-culture experiments to see whether the survival of prey bacteria (i.e., *N*. *cinerea*, *E*. *coli*, and *L*. *crispatus*) was affected in the presence of wild-type *N*. *gonorrhoeae* or an isogenic mutant unable to express Naps. We constructed a markerless *N*. *gonorrhoeae* FA1090 Δ*napRABC* mutant (eliminating all Nap peptides) with a pheS* counter-selection marker [26] to avoid fitness costs associated with antibiotic resistance cassettes (**S3 Fig**). After 3 and 24 h of co-culturing prey and *N*. *gonorrhoeae* at a 1:1 ratio, we recovered prey strains by plating to selective media. No significant difference could be observed in the recovery of prey strains in the presence of wild-type or Δ*napRABC N*. *gonorrhoeae* (multiple paired *t* tests, *n* = 3, **S4 Fig**). Taken together, these results demonstrate that Naps do not have detectable antimicrobial activity.

### Naps mediate *N*. *gonorrhoeae* autolysis

**Fig 2:**
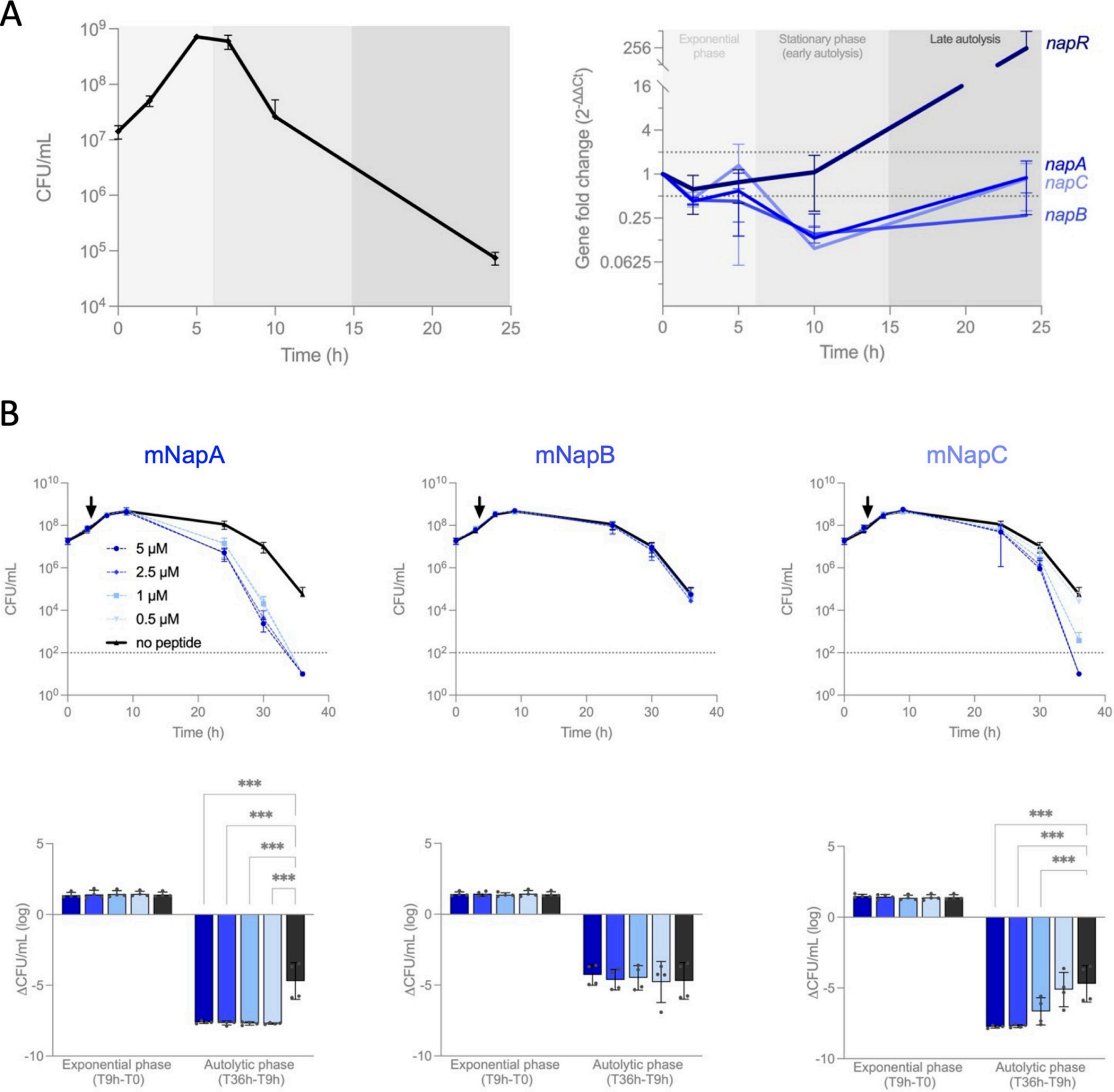
Regulation of the *nap* island and the effect of mNaps on bacterial survival. **(A)** RT-qPCR of genes in the *nap* island from *N*. *gonorrhoeae* FA1090 grown in GW liquid cultures. Samples were taken from flasks at different times (CFU, left panel) and mRNA levels of target genes determined by RT-qPCR (right panel). Gene expression results were normalised to samples at T0. **(B)** Bacteria were grown in 96-well plates in protein-free spermidine-free GW medium for 3 h before mNaps were added at various concentrations (arrow). At time points indicated, bacterial viability was determined by plating to solid media and incubation overnight. CFU were counted (dotted lines, limit of detection). Bottom panels represent the same data but grouped according to the growth phase (exponential and autolytic). Error bars, SD, two-way ANOVA (*n* = 4; *p* ≥ 0.033, not significant, not represented; *p* < 0.001, ***). The data underlying this figure can be found in S3 Data.

To better understand the function of the Naps, we examined the regulation of genes encoding *nap* island peptides as the expression of many microcins and bacteriocins is regulated during growth [13] (**Figs 2A** and **S5A**). The mRNA levels of genes encoding the nap peptides was measured by RT-qPCR of bacteria grown in liquid media over 24 h. The mRNA levels for *napA*, *napB*, *napC*, and *napR* were lowest in the early stationary phase of growth, then rose to their highest levels in the late autolytic phase. The similar gene expression profile of *napA*, *napB*, and *napC* suggests that they are organised in operon. This is consistent with the absence of a predicted promoter directly upstream of *napB* (S**5B Fig**) and the detection of a transcript overlapping all 3 genes in *N*. *gonorrhoeae* MS11 [27]. The *nap* locus of *N*. *gonorrhoeae* FA1090 also harbours Correia elements (CEs) in the *napR*/*napA* and *napI*/*napH* intergenic regions containing potential promoters [28] (**Figs 1A** and S**5B**); these CEs harbour integration host factor (IHF) binding sites (S**5C Fig**), which can influence transcriptional regulation [29].

As *nap* mRNA levels were elevated during the late stationary phase, we considered whether the Naps are involved in PCD. Gonococcal autolysis involves initial peptidoglycan remodelling by LtgA and other enzymes [4,30], followed by cell lysis. As polyamines can increase the resistance of bacteria to cationic peptides [31,32], we assessed the activity of mNaps on gonococcal survival in protein/spermidine-free media. mNaps were added to *N*. *gonorrhoeae* at the mid-log phase, and survival examined during the stationary phase until autolysis occurred [33]. *N*. *gonorrhoeae* was grown for 3 h in 96-well plates from a starting OD_600_ of 0.25, then exposed to mNaps. While mNaps had no effect on survival of gonococci during the exponential phase of growth, micromolar concentrations of mNapA and mNapC significantly reduced survival when bacteria reached the late stationary phase of growth (**Fig 2B**, *p* < 0.001 versus no peptide). Specifically, mNaps had no effect on *N*. *cinerea* at any stage of growth (**S6A Fig**), while scrambled versions of mNapA and mNapC (mNapA_SCR_ and mNapC_SCR_, respectively) did not affect survival of *N*. *gonorrhoeae* (**S6B Fig**). We also checked whether mNaps interacted with each other in this assay. Mixtures of Naps were tested on *N*. *gonorrhoeae* and results compared with effect of individual mNaps. There was no evidence of antagonism or synergy between the Naps in these conditions (**S6C Fig**).

**Fig 3:**
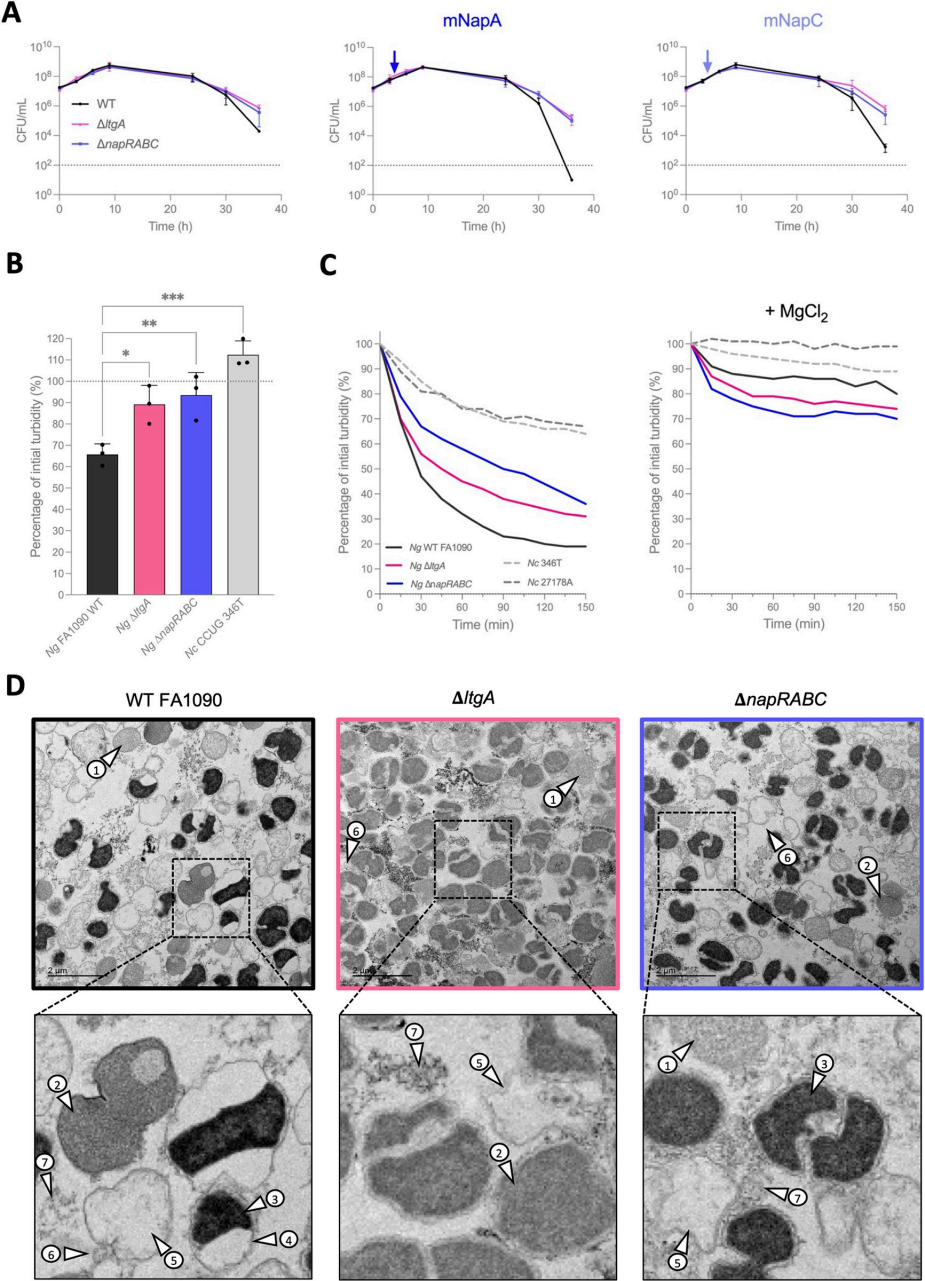
The *nap* island is involved in autolysis. **(A)** Long-term survival of wild-type *N*. *gonorrhoeae* FA1090 and the Δ*napRABC* mutant in the presence (arrow) or absence of mNaps (5 μm). The markerless *ltgA* deletion mutant was a control for a strain with reduced autolysis. Dotted lines, limit of detection. **(B)** Autolysis in rich medium. Bacteria were streaked on plates, grown overnight then resuspended in GCB at OD_540_ of ~0.3 and left in cuvettes. After 24 h at room temperature, bacteria were gently resuspended and the OD_540_ measured. One-way ANOVA with Dunnett’s multiple comparison against the wild-type strain (*n* = 3; *p* < 0.033, *; *p* < 0.002, **; *p* < 0.001, ***). The data underlying this figure can be found in S4 Data. **(C)** Autolysis in HEPES buffer without (left panel) or with (right panel) 10 mM MgCl_2_. A representative experiment is shown (last replicate is in the corresponding Suppl. source data files). **(D)** Transmission electron microscopy images from bacteria in HEPES (T = 75 min). Some wild-type bacteria appear normal (indicated with arrow numbered 1), with many others having a dense (arrow 2) or very dense (arrow 3) cytoplasm, periplasmic expansions (arrow 4), or appearing as ghost cells (arrow 5). Extracellular vesicles (arrow 6) and released cellular content (arrow 7) are also visible. Periplasmic expansions are not observable in the Δ*ltgA* or Δ*napRABC* strains. Cytoplasm condensation appears more homogenous in the Δ*ltgA* mutant. The images underlying this figure can be found in https://doi.org/10.6084/m9.figshare.28077749.v1.

As a control for autolysis, we next constructed a markerless *N*. *gonorrhoeae* FA1090 Δ*ltgA* mutant; *ltgA* is involved in the first step of gonococcal autolysis [4]. Importantly, both the Δ*napRABC* and Δ*ltgA* mutants displayed increased survival during the late stationary/autolytic phase of growth compared with wild-type bacteria, whether in presence or absence of exogenously added mNaps (**Fig 3A**, at t, 36 h, *p* = 0.002, ** for Δ*ltgA*, and *p* = 0.03, * for Δ*napRABC* in the absence of Naps, and *p* < 0.001, *** for both strains in the presence of mNapA or mNapC, two-way ANOVA), consistent with the Naps contributing to autolysis.

To establish whether the *nap* island directly contributes to PCD, we examined wild-type, Δ*napRABC*, and Δ*ltgA N*. *gonorrhoeae* in 2 independent assays of autolysis; *N*. *cinerea* was included as a control. First, we assessed changes in the OD_540_ of strains incubated overnight in liquid GCB (**Fig 3B**); a reduction in OD provides a measure of autolysis [34,35]. While the OD_540_ of wild-type bacteria fell overnight by 34.3% (±4.9, indicating autolysis), the OD_540_ of the Δ*ltgA* and Δ*napRABC* mutants only dropped by 10.8% (±8.9) and 6.5% (±10.6), respectively (**Fig 3B**, *p* < 0.033 and 0.002 versus the wild-type strain), indicating that both mutants exhibit reduced autolysis. In contrast, the OD_540_ of *N*. *cinerea* increased over this time (**Fig 3B**, +12.4% ± 4), consistent with the lack of PCD in this species. The rate of autolysis of these strains was also estimated by following the OD_540_ of bacteria incubated in HEPES, as starvation in this buffer can induce autolysis [5]. Again, the Δ*ltgA* and Δ*napRABC* mutants exhibited decreased rates of autolysis compared with wild-type bacteria (**Fig 3C**), as shown by the turbidity of cultures at the mid-time point (75 min, *p* < 0.033 and *p* < 0.002, respectively, S**7A Fig**). Autolysis of 2 strains of *N*. *cinerea* was also significantly less than *N*. *gonorrhoeae* in this assay (**Figs 3C** and **S7C**). Consistent with the ability of divalent cations to impair autolysis [34], addition of MgCl_2_ (final concentration, 10 mM) markedly decreased autolysis (**Fig 3C**) and abolished any significant difference in autolysis between the wild-type, Δ*ltgA* and Δ*napRABC* strains (S**7B Fig**). To understand the role of NapI during autolysis, we attempted to generate a *napI* mutant by replacing its open reading frame with a kanamycin resistance cassette. Despite multiple attempts, this was unsuccessful in wild-type bacteria presumably because of the toxicity of Naps in the absence of their immunity protein. Consistent with this, we were able to generate a *napI* mutant in the Δ*napRABC* strain. The *napI* mutant was incubated in HEPES and the OD_540_ measured over time. Surprisingly, the Δ*napRABCI* mutant was even more resistant to autolysis than the Δ*napRABC* strain (**S7A Fig**), suggesting that NapI might contribute to autolysis or bacterial fitness in absence of other Naps.

Finally, we examined the ultrastructure of the wild-type, Δ*ltgA*, and Δ*napRABC* strains by transmission electron microscopy (TEM) after incubation in HEPES buffer to induce autolysis. Before resuspension in HEPES, the strains were indistinguishable with clear nucleoids visible (**S8 Fig**). After 75 min, wild-type bacteria showed a mix of phenotypes (**Fig 3D**), some with dense cytoplasm, while other cells displayed an expanded periplasm, or were empty “ghost” cells with evidence of nearby debris. The cytoplasm of the Δ*ltgA* mutant appeared less dense than wild-type bacteria, with no visible periplasmic expansions (**Fig 3D**). Similarly, the Δ*napRABC* mutant lacked periplasmic expansions (**Fig 3D**), with many cells having aberrant shapes (**Fig 3D)**. Based on the OD_540_ of bacteria in buffer, both mutants do undergo autolysis. However, TEM images indicate that cell death proceeds differently for the Δ*ltgA* and Δ*napRABC* mutants based on differences seen in the expansion of their periplasm. Taken together, our data provide evidence that the Naps contribute directly to autolysis of *N*. *gonorrhoeae*.

### NapC induces host erythrocyte lysis

**Fig 4:**
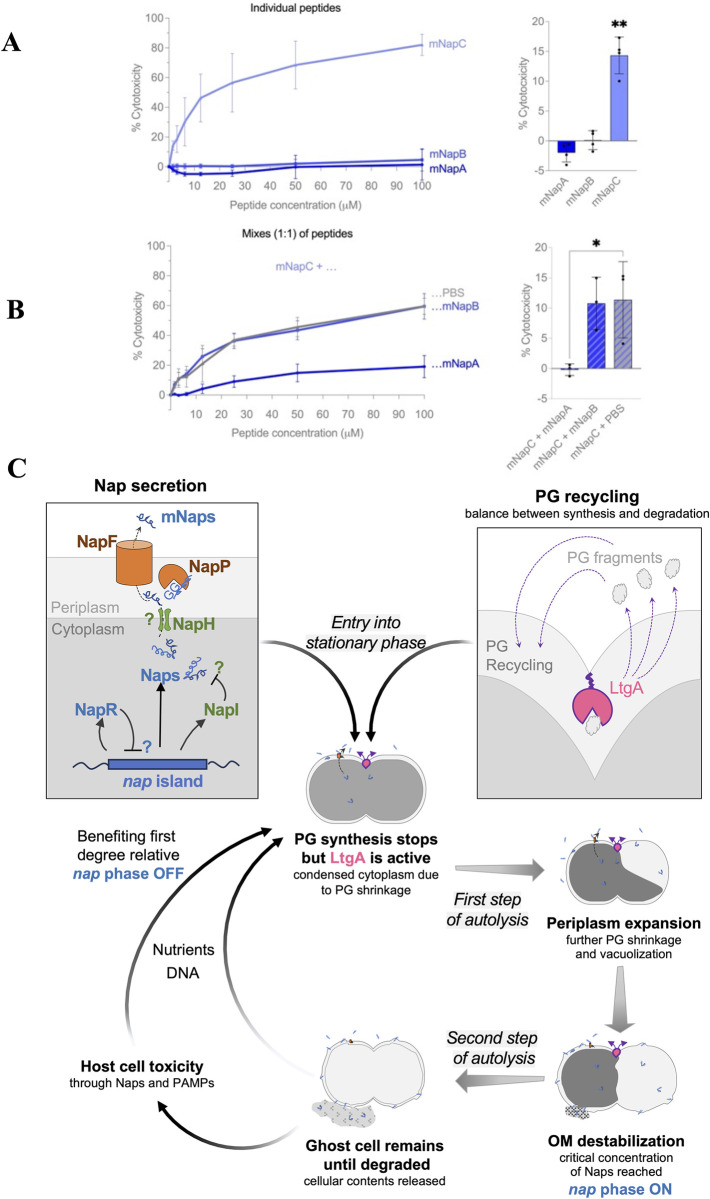
Effect of Naps on human red blood cells and proposed model. **(A)** Different concentrations of mNaps were added to human RBCs for 1 h and the OD_414_ measured. Data were normalised to cytotoxicity with melittin (100%), or PBS (0%). Right panel, cytotoxicity induced by 1.56 μm of each mNap (*n* = 4; *p* < 0.002, **). **(B)** mNapA or mNapB were mixed with mNapC at a 1:1 ratio and cytotoxicity measured. Right panel, results with 3.15 μm of each peptide, *n* = 3, *p* < 0.033, *, one-way ANOVA with Dunnet’s multiple comparisons. The data underlying this figure can be found in S5 Data. **(C)** Proposed model of gonococcal autolysis. Peptidoglycan (PG) remodelling enzymes, including LtgA (pink) which localises at the septum, will release PG fragments that are recycled (purple arrows). When bacteria reach stationary phase, PG synthesis stops, but LtgA remains active, leading to the condensation of the cytoplasm (Fig 3D). Meanwhile, Naps are processed and secreted by NapP and NapF (orange), respectively, leading to their accumulation in the extracellular environment. Vacuolation is triggered, potentially through the action of inner membrane destabilising actors such as holins/toxins. Eventually, the outer membrane (OM) breaks when a critical concentration of mNaps (blue) is reached, leading to release of cellular contents and the appearance of ghost cells. For diplococci, cells undergoing PCD will benefit first-degree relatives by direct and indirect mechanisms.

As the *nap* island is largely limited to the gonococcus and meningococcus which can elicit marked inflammatory responses [36], we examined whether the mNaps are toxic to host cells. mNaps were tested for their ability to lyse human RBCs over 1 h at 37°C. Interestingly, mNapC had marked haemolytic activity even at low concentrations (<2 μM, **Fig 4A**), while neither mNapA or mNapB were toxic. To check for interactions between the peptides, we added combinations of mNaps in a 1:1 ratio to cells. Surprisingly, mNapA protected RBC from lysis by mNapC (**Fig 4B**). To examine whether mNaps are toxic to other host cells, we also measured the release of lactate dehydrogenase (LDH) by THP-1-derived macrophages following exposure to 1 or 5 μm of each mNap (**S9 Fig**). No cytotoxicity was detected against THP-1 cells with any mNap, suggesting that their effect is cell-type specific.

## Discussion

Many bacteria undergo PCD when subjected to specific environmental stresses such as nutrient deprivation and oxidative damage [37]. This can be mediated by toxin:antitoxin systems, which can also limit the spread of bacteriophage in a population through a process known as abortive infection [37]. In contrast, notably few bacterial species undergo autolysis as a part of their natural life cycle when they enter the stationary phase of growth. Among bacteria with the potential to cause invasive disease, PCD is a feature of the gonococcus, meningococcus, and pneumococcus. These species share the characteristics of being diplococci that are naturally transformable inhabitants of mucosal surfaces in humans. These features are likely to have contributed to autolysis being an adaptive trait in this specific subset of bacteria. We show that the invasive species of *Neisseria* have acquired a 3.4 kb island which is necessary for their ability to undergo PCD. The success of the *nap* island as a selfish genetic element is evidenced by its almost ubiquitous distribution in *N*. *gonorrhoeae* and *N*. *meningitidis*, indicating that the *nap* island and thence autolysis promotes the success of these bacteria at mucosal surfaces and their invasive potential.

For the gram-positive bacterium *S*. *pneumoniae*, the major autolysin LytA is responsible for degradation of cell wall peptidoglycan which is sufficient to cause cell lysis [38]. The situation is more complex for gram-negative bacteria, which possess 2 membranes. Autolysis of pathogenic *Neisseria* was described over a century ago [3,39]. The first step of gonococcal PCD is peptidoglycan remodelling which depends on the activity of lytic transglycosylases such as LtgA [4,5,30,34]. However, the mechanisms underlying subsequent steps in this process have remained obscure. However, it was known that blocking protein synthesis suppresses autolysis [40] without impeding peptidoglycan hydrolysis [5], suggesting that other proteins are involved in autolysis. Here, we demonstrate that Naps mediate the second step of gonococcal autolysis. We show that addition of mNapA and mNapC reduce bacterial survival specifically during the stationary phase of growth when autolysis occurs. In addition, the *napRABC* mutant lacking all Naps displayed reduced autolysis compared to the wild-type bacteria. As well as promoting PCD, NapC can be toxic to host RBCs. The peptides are encoded by the *nap* island, a small horizontally acquired genetic element predominantly found in species of *Neisseria* which have the capacity to cause invasive disease.

Aside from mediating the release of bacterial PAMPs on death, mNaps might contribute directly to host:pathogen interactions by being toxic to human cells. mNapC can mediate lysis of RBCs at micromolar concentrations; this is counteracted by mNapA, indicating that the interplay of mNaps influence their effect on human cells. In our work, we could not attribute a function to mNapB. One possibility is that NapB does not to regulate autolysis but instead influences other aspects of bacterial life. For example, in *S*. *pneumoniae*, competence and fratricide lysis have been functionally linked, since an early competence gene encodes an immunity protein against their own lysins [41]. Since a functional link between the 2 phenomena might also exist in *N*. *gonorrhoeae* [42], it might be worth investigating the effect of NapB on competence. Additionally, gram-positive bacteria can also produce multiple bacteriocins; in lactic acid bacteria, the diversity of peptides offers an advantage in distinct environments [43]. Similarly, *N*. *gonorrhoeae* might possess multiple Naps to respond to different environmental cues to leading to PCD +/− toxicity of host cells. Considering that some killing mechanisms require direct bacterial contact with target cells [44], it is also possible that gonococcal Naps have further toxic effects on host cells in the presence of bacteria.

Further work is needed to understand the function of the proteins encoded by the *nap* island. Nevertheless, we propose a model for the role of Naps during autolysis (**Fig 4C**). *N*. *gonorrhoeae* PCD starts with re-modelling of peptidoglycan. Cationic Naps produced in the cytoplasm as immature pre-peptides are cleaved in the periplasm and secreted by NapP and NapF, respectively. The role of the other genes on the *nap* island is more speculative as they are not always found in *nap* islands in other species of *Neisseria*. NapH has features of an inner membrane holin, which allow escape of phages from host cells [45], so might be involved in transporting Naps across the inner membrane. NapR (with a predicted DNA binding domain) could regulate genes on the *nap* island, while the function of NapI (which has some homology to the immunity protein LagC [18]) could prevent self-intoxication by the cationic Naps.

PCD has several potential benefits to bystander bacteria, such as provision of nutrients [46], enhancing biofilm formation, and the release of DNA to promote genetic diversification of naturally competent bacteria, such as the pathogenic *Neisseria*. In addition, bacterial lysis could perturb host cells to release nutrients, or alter local inflammatory responses [4]. Lysed RBCs would also release iron, which could help bacteria circumvent nutritional immunity [47]. Importantly, autolysis is also concomitant with the release of bacterial phospholipids [35,48] and peptidoglycan fragments [40,49], which can limit bacterial growth [50] and reduce host innate immune signalling [51], respectively. In *S*. *pneumoniae*, the extent of autolysis is correlated with hyper-virulence [8], while the acquisition of horizontally acquired genomic islands in *N*. *meningitidis* are associated with invasive capacity of strains [52].

There is a debate over how autolysis evolves in single-celled organisms [37], as PCD is literally a dead-end for a bacterium. Interestingly, NapR and NapC are likely to be phase variable, based on different lengths of homopolymeric tracts in their open reading frames. Based on sequences of over 10,000 gonococcal isolates in PubMLST, *napC* seems to be mainly OFF (86% of sequenced *N*. *gonorrhoeae* strains, *n* = 7,773/10,854) with an early stop codon preventing the production of the C-terminal cationic amino acids. Phase variation has implications about how the *nap* island and PCD could be beneficial to a clonal bacterial population [53], and would generate different subpopulations of gonococci, with some bacteria refractory to PCD with other bacteria undergoing altruistic cell death. A bet-hedging strategy, with bacteria switching between 2 distinct phenotypes, could explain how autolysis and altruism evolved in the gonococcus [37]. It is noteworthy that PCD, which is rare among prokaryotes, has evolved through distinct mechanisms in pathogenic diplococci, which are naturally transformable. The release of DNA on autolysis could allow the transfer of beneficial traits between siblings. PCD might be particularly beneficial for diplococci with a dying cell benefitting their first-degree relatives in immediate proximity, i.e., their nearest and dearest (**Fig 4C**).

In summary, our study sheds light on PCD in invasive *Neisseria*, a fundamental process relevant for gonococcal cell biology; the *nap* island encodes NapC which has dual activity, triggering autolysis in addition to death of host cells. As well as the direct effect of the Naps, bacterial suicide might also trigger local and systemic inflammation through the release of PAMPs. Thus, the acquisition of the *nap* island and its success in *N*. *gonorrhoeae* and *N*. *meningitidis* might have been an important step in the emergence of these species as invasive pathogens, by enabling them to undergo autolysis to manipulate immune responses and the local environment.

## Materials and methods

### Bacterial strains and growth

Bacterial strains used in this study are listed in **S3 Table**. *N*. *gonorrhoeae* and *N*. *cinerea* were grown on GCB agar plates (1.5% wt./vol. proteose peptone number 3, Becton Dickinson, 0.1% starch, 0.4% K_2_HPO_4_, 0.1% KH_2_PO_4_, 0.5% NaCl, 1% Vitox, Oxoid, 1.5% agar Oxoid) [54] at 37°C with 5% CO_2_. *L*. *crispatus* was grown on MRS agar plates (ATCC medium 416) and incubated at 37°C with 5% CO_2_ for about 36 h. *E*. *coli* was grown on lysogeny broth (LB) agar plates at 37°C.

### Generation of deletion mutants

All primers are shown in **S3 Table**. For allelic replacement, overlap PCR was performed to obtain resistance cassettes (*aph(3)-I* or *ermC*, for kanamycin or erythromycin resistance, respectively) flanked by regions (approximately 1,000 bp) surrounding the target gene. Briefly, overlap PCR consisted in mixing 3 PCR products (“upstream PCR 1” including the START codon of the targeted gene; “downstream PCR 2” including the STOP codon of the targeted gene; “resistance cassette PCR 3”) in equal ratios. For markerless deletions, 2 distinct overlap PCR products were generated: (a) a PCR product consisting of homologous regions (“upstream PCR 1” and “downstream PCR 2”) flanking selection and counterselection cassettes controlled by a constitutive promoter (“*p*_*opaB*_-kanR-pheS* PCR 3”, **S3 Fig**); (b) a markerless PCR product consisting of homologous regions directly bound to each other (“upstream PCR 1” and “downstream PCR 2”). *N*. *gonorrhoeae* was transformed as previously [54]. For selection erythromycin (0.5 μg ml^−1^), kanamycin (80 μg ml^−1^), or 4CP (8 mM) were added to media. Individual transformants were screened by PCR and confirmed by sequencing.

### Bioinformatic analysis

The *nap* island was identified by MaGe [17] by comparing synteny maps between the reference *N*. *gonorrhoeae* FA1090 and *N*. *gonorrhoeae* NCCP11945, *N*. *gonorrhoeae* FA6140, *N*. *gonorrhoeae* 35/02, *N*. *gonorrhoeae* PID24-1, *N*. *meningitidis* 053442, *N*. *meningitidis* FAM18, *N*. *meningitidis* MC58, *Neisseria lactamica* ATCC 23970, *N*. *cinerea* ATCC 14685, *Neisseria flavescens* NRL30031/H210, *N*. *flavescens* SK114, *Neisseria subflava* NJ9703, *Neisseria sicca* ATCC 29256 and *Neisseria mucosa* ATCC 25996. Using PubMLST [20], the ORF loci and alleles associated to the *nap* island were manually curated, as described in https://bigsdb.readthedocs.io/en/latest/curator_guide.html and https://www.youtube.com/watch?v=09g5YdrCtDc. Briefly, using the *Neisseria* isolates database curator’s interface, the “sequence tags scan” tool allowed us to retrieve alleles in given batches of isolates (see below for isolates selection criteria). New alleles were then validated through sequence alignment and added to the database using *Neisseria* typing database curator’s interface, “sequences (batch) add” tool. This process was repeated until at least 93.5% of the selected isolates had an allele number attributed to each *nap* gene. To determine gene conservation (**Fig 1C**), manual strain selection from the isolate collection of each species on PubMLST was done using the following criteria: a complete rMLST to confirm the species, and the number of contigs <500. Gene presence was assessed using “Analysis/Gene Presence” for the different NEIS loci, with pre-set parameters (Min % identity: 70, Min % alignment: 50, BLASTN word size: 20). Gene conservation data are detailed in **S2 Table.**

### Reverse transcription followed by quantitative PCR

Bacteria were grown in 50 ml of protein- and spermidine-free GW medium [55] in vented flasks (Corning, 431144), by inoculation at OD_600nm_ 0.025 from bacteria grown on GCB plates (T_0_). RNA was purified from 2 ml of culture pelleted for 4 min at 4,500 rpm and resuspended in TRIzol for 5 min then frozen at −80°C. Tubes were then thawed on ice and mixed with 200 μl of chloroform by vigorous shaking of tubes. After 2 to 3 min incubation at room temperature, tubes were centrifuged at 4°C for 15 min at 12,000 *x g* and the aqueous phase transferred to 500 μl of ice-cold isopropanol. Tubes were incubated overnight at −20°C, then centrifuged at 4°C for 30 min at 20,000 *x g*, and pellets were washed with 75% ice cold ethanol, before being resuspended in 80 μl diethylpyrocarbonate-treated H_2_O. Samples were treated with ezDNase (Invitrogen) and first strand cDNA synthesis was performed with SuperScript IV reverse transcriptase (RT) (Invitrogen) according to manufacturer’s instructions. Note that to obtain specific cDNA, reverse primers (available in **S3 Table**) were used (alongside the one for the housekeeping gene *recA*), and a no-RT control was done in parallel. Samples were treated with RNase H for 20 min at 37°C before qPCR was performed using SYBR Green PCR master mix (Applied Biosystems). Primer efficiency was evaluated using serial dilutions to generate a standard curve from PCR products (pre-screen) and mixes of cDNA (to reflect real qPCR conditions). The slopes of standard curves and efficiency values for each primer pair was calculated. StepONEPlus real-time PCR software was used to collect RT-qPCR data and the ΔΔCt method [56] was used to analyse the data, using *recA* Ct values as reference gene and T_0_ Ct values as reference condition for **Fig 2A**. Experiments were done with at least 3 biological replicates, each time with duplicate or triplicate technical repeats.

### mNap synthesis

mNaps were synthesised and subjected to HPLC/mass spectrometry by Isca Biochemicals (UK). Note that mNapA was acetylated on its N-terminal side to increase stability. Peptides were diluted in dezionized water supplemented with 0.001% trifluoroacetic acid at 10 mg/ml, aliquoted in 10 μl fractions in Protein Lo-bind tubes (Eppendorf), snap frozen with liquid nitrogen, and stocked at −80°C. Each aliquot was only used once and immediately upon thawing. Batches of peptides were not stocked for longer than 6 months to avoid loss of activity.

### Disk diffusion and MBC assays

Bacteria were freshly harvested from plates and resuspended in GW medium [55] at 10^8^ bacteria/ml then spread on agar plates. *L*. *crispatus* was plated on MRS agar plates, while all other bacteria were plated on GW agar plates, prepared by mixing (50:50) 2×-concentrated liquid GW medium (filtered) with warm autoclaved 2% agar in water. Plates were allowed to dry for 10 to 15 min, then 6 mm Whatman paper disks soaked with 10 μl of peptide (50 μm) were added. Polymyxin B (50 μm) and water were used as controls. Plates were incubated for 24 to 48 h at 37°C and 5% CO_2_ as required. The efficacy of compounds was assessed by the presence of a zone of growth inhibition around disks.

All bacteria were harvested from plates and resuspended in FB medium [57]. Peptides were prepared in FB medium as 2×-concentrated solutions at 512 μg/ml and 2-fold dilutions (to 10 μg/ml) were prepared in untreated U-bottom polypropylene 96-well plates (Corning, 3879) to 50 μl per well. Penicillin G and polymyxin B were used as controls. Bacteria (50 μl of 10^5^ CFU/ml) were added to each well, then incubated at 37°C with 5% CO_2_ with shaking at 180 rpm for 24 h, before spotting 10 μl of each well to plates. After 24 h of incubation, MBC values were attributed to the lowest concentration which gave no growth.

### Co-culture assays

Bacteria were harvested from plates, resuspended in FB medium [57], and diluted to 10^5^ CFU (colony-forming unit)/ml. Aliquots of 250 μl of prey bacteria were mixed with 250 μl of either wild-type *N*. *gonorrhoeae* FA1090, or the Δ*napRABC* mutant, or FB medium alone. Cultures were incubated at 37°C with 5% CO_2_ with shaking at 180 rpm for 3 and 24 h, before plating to selective agar plates; for *N*. *cinerea*, *E*. *coli*, and *L*. *crispatus*, media were GCB plates supplemented with 0.01% Congo Red [58], LB agar plates, and MRS agar plates, respectively. The number of prey bacteria were normalised to control wells without added gonococci.

### Growth curves

Bacteria were resuspended in liquid medium at OD_600_ 0.025, and 100 μl added to wells of a flat-bottom 96-well plate (Greiner, 655161), and the OD_600_ monitored with a plate reader (BMG LabTech) at 37°C in 5 % CO_2_ with shaking at 200 rpm. Alternatively, bacteria were resuspended in protein-free spermidine-free GW medium at an OD_600_ of 0.025, then 90 μl added to wells of untreated U-bottom polypropylene plates (Corning, 3879). After 3 h, 10 μl of peptide was added. Bacterial survival was measured by plating on chocolate agar plates with 5% defibrinated horse blood (E&O labs, PP0100).

### Autolysis assays

Bacteria were resuspended in 1.2 ml liquid GCB at OD_540_ of ~0.3, then left in cuvettes for 24 h at room temperature (21°C), gently resuspended by pipetting before the OD_540_ was measured again.

Additionally, bacteria were grown in liquid GCB (OD_600_ of ~0.025) for ~4 h to reach mid-exponential growth, pelleted at 4,500 *x g* for 5 min and resuspended in 700 μl HEPES buffer (50 mM, pH 8.5) to an OD_450_ of ~0.3 per ml. Aliquots were added to cuvettes prefilled with 300 μl of HEPES buffer (50 mM, pH 8.5). Just after mixing in pre-filled cuvettes, the OD_450_ was taken (T_0_, 100%), then again at regular intervals after gentle resuspending. Values are given in percent of initial turbidity.

### Transmission electron microscopy

Samples were prepared as when measuring autolysis in buffer. After 75 min, bacteria were recovered from cuvettes and pelleted at 4,500 G for 5 min before being resuspended in fixation buffer (2.5% glutaraldehyde, 2% formaldehyde, 0.1 M PIPES buffer (pH 7.2)) and left at room temperature for 1 h then stored at 4°C. After fixation, samples were extensively washed in buffer then pelleted, embedded in agarose and dissected into ≤1 mm^3^ cubes. Samples were treated with 50 mM glycine in buffer for 15 min then washed ahead of secondary fixation for 1 h at 4°C in 1% osmium tetroxide and 1.5% potassium ferrocyanide in buffer. Samples were washed extensively in water and stained overnight in 0.5% uranyl acetate (aq.) at 4°C. The following day, samples were washed in water then dehydrated step-wise in a series of 30%, 50%, 70%, 80%, 90%, 95%, and 100% ethanol. The dehydrated samples were incubated in a 25% solution of low viscosity resin (Agar) diluted in ethanol, then in a 50% solution overnight. Samples were further infiltrated in 75% and then extensively in 100% resin ahead of embedding and polymerisation. Sections of 90 nm were cut from polymerised sample blocks using a Leica UC7 ultramicrotome, post-stained with lead citrate and imaged on a Thermo Fisher Tecnai T12 TEM at 120 keV (with Gatan OneView CMOS camera).

### Haemolysis assay

Human blood (1 ml; K2EDTA; Cambridge Bioscience, United Kingdom) was washed 3 times with 4 ml PBS prior to centrifugation at 700 *× g* for 8 min as previously [59]; erythrocytes were pelleted at 1,000 *× g* for 10 min, and diluted to 0.5% v/v. Peptides were added to 96-well V-bottomed polypropylene plates at a starting concentration of 100 μm. Mellitin (2.5 μm) and PBS were added as positive and negative controls. Erythrocytes were incubated at 37°C for 1 h, pelleted at 1,000 *× g* for 10 min; the OD_415 nm_ of supernatants (60 μl) was read, and results normalised to the controls.

### LDH release assay

THP-1 Dual cells (InvivoGen) were maintained in RPMI1640 medium supplemented with 10% heat inactivated FBS, 25 mM HEPES buffer, 100 U/ml penicillin, and 100 μg/ml streptomycin at 37°C, 5% CO_2_. Cells were differentiated to monocyte-derived macrophages (MDMs) in 96 plates (10^5^ cells per well in 200 μl media) using 50 nM of phorbol 12-myristate 13-acetate (PMA, MP Biomedicals) for 3 h, followed by 3 days in complete media. When required, cells were activated with LPS from *E*. *coli* O111:B4 (100 ng/ml, Sigma) for 1 h before being exposed to mNaps.

CytoTox 96 Non-Radioactive Cytotoxicity Assay (Promega) was used to quantify cell death according to the manufacturer’s protocol. After 6 h, 50 μl of culture supernatant was added to an equal volume of Cytotox 96 reagent. Samples were incubated at room temperature protected from light for 30 min. Then, the OD_490_ using a microplate reader (BMG Labtech PHERAstar FS) and results normalised to controls.

### Statistical analysis

Statistical analyses were performed on GraphPad Prism version 10.0.0, using tests described.

## Supporting information

S1 Fig Supplementary information regarding genes of the *nap* island in *Neisseria gonorrhoeae* FA1090. DNA and amino acid sequences are given for each *nap* genes, as well as predicted alpha-fold structures and extra information, such as C39 peptidase cleaving sites, phase variable sequences based on available genomic data (details available in S1 Table) or gene alignments. Note that the alignment between *napI* and the Lactococcin-G immunity protein *lagC* was performed after the serendipitous observation that NapP was annotated as homolog to the “Lactococcin-G-processing and transport ATP-binding protein” LagD from *Lactococcus lactis* both in the genome of *N*. *gonorrhoeae* FA19 (GenBank accession no. CP012026.1, locus tag: VT05_00181) and *N*. *gonorrhoeae* 35/02 (GenBank accession no. CP012028.1, locus tag: WX61_01768).
(PDF)

S2 Fig Nap island organisation in *Neisseria meningitidis (Nm)* MC58, *Nm* FAM18, *Neisseria lactamica (Nl)* 020–06, and *Neisseria cinerea* (*Nc*) NCTC10294. In both *Nm* and *Nl* strains, NapF sequences correspond to the long NEIS0907 allele, which is found in another loci in *N*. *gonorrhoeae (Ng)* (under the gene name NGO_0166 in *Ng* FA1090). In *Nm*, chromosomal rearrangements led to the fusion between the 2 NEIS0907-containing loci. Genes in purple correspond to those homologous to the NGO_0166-containing locus in *Ng* FA1090. In *Nl*, the presence of the long NEIS0907 allele correlates with the loss of *napA*. Regarding *napB*, due to the presence of an earlier start codon, it is present as a longer allelic form than in *Nm* or *Ng*. As for *napI*, it is slightly shorter in *Nl* than in *Ng*. Note that an extra gene is represented in black in the locus of *Nl* (NLA_13780, function unknown); a homolog sequence exists in *Ng* FA1090 but the start codon is not present. As shown in Fig 1C, the locus is absent in *Nc*. Instead, 3 genes of unknown function (light grey) are present between the flanking NEIS0794 and NEIS0808. ⁑, C39 peptidase predicted cleavage site. Note that for NapB in *Nl*, ⁑ was not positioned as alternative GG-sites might be at play. Sequences were manually annotated on Snap Gene viewer.
(PDF)

S3 Fig Construction and characterisation of deletion mutants in *N*. *gonorrhoeae*. (A) Growth curves in GCB medium. Markerless deletion strains (plain lines, ^ML^) are compared to resistant marker strains (dotted lines, ^K^ for kanamycin resistance marker and ^E^ for erythromycin resistance marker). Standard deviation are shown in lighter colours (*n* = 9). The data underlying this figure can be found in S6 Data. (B) Schematic representation of the classical method to generate deletion mutants in *N*. *gonorrhoeae*. Briefly, an overlap PCR product is generated from upstream region (PCR 1), downstream region (PCR 2), and resistance cassette marker (PCR 3) amplifications. The Overlap PCR product is then used for natural transformation into *N*. *gonorrhoeae* and the mutant strain is selected based on the acquired antibiotic resistance. (C) Schematic representation of the markerless method. Briefly, 2 overlap PCR products are generated from upstream region (PCR 1) and downstream region (PCR 2) as well as, for the first product only, an endogenous promoter (p_*opaB*_) followed by section/counter-selection markers (PCR 3) amplifications. Note that the pheS* sequence used here was amplified from the genome of *N*. *gonorrhoeae* FA1090 itself and point mutations (* = T275S and A318G) were then introduced by overlap PCR. The markerless method consists in first selecting a recombinant colony through kanamycin resistance selection, and secondly, by transforming this colony with markerless overlap PCR product to allow the counter-selection of the pheS* marker.
(PDF)

S4 Fig Co-culture of prey bacteria with *N*. *gonorrhoeae*. Prey bacteria were incubated alongside either wild-type *N*. *gonorrhoeae* (WT FA1090, black bars) or the Δ*napRABC* strain (blue bars) for 3 or 24 h in FB medium at a 1:1 ratio. Prey bacteria were then recovered selectively on agar plates and CFU/ml were counted. Data were normalised against the recovery of prey bacteria grown without the gonococcus (100%). The data underlying this figure can be found in S7 Data. Multiple paired *t* test were performed between each pair (WT vs. Δ*napRABC*) with no significant difference in their survival (*n* = 3, error bars, SD).
(PDF)

S5 Fig Gene expression of the *nap* island in *N*. *gonorrhoeae*. (A) RT-qPCR on all genes of the *nap* locus of strain FA1090. Samples were recovered from GW liquid cultures in flasks at different time points, and gene expression was normalised based on samples recovered from plates and resuspended in GW (T0). Error bars represent standard deviation from the mean (*n* = 3–4). One-way ANOVA was performed on data with minimum 2-fold change (dotted lines) compared to T0 (*p* < 0.033, *; *p* < 0.002, **; *p* < 0.001, ***). Growth curves were performed in parallel (right bottom panel). The data underlying this figure can be found in S8 Data. (B) Predicted ribosome binding sites (RBS) and promoter regions (grey arrows) in the *nap* locus of strain FA1090. RBS were manually annotated based on the presence of a GGA enriched sequence −3 to −10 of an ATG start codon; promoter regions predicted by BPROM (http://softberry.com). (C, D) Pink arrows represent repeats of Correia elements (CE), while blue boxes show integration host factor (IHF) binding sites, as defined previously (10.1016/s0378-1119(01)00725-9 and 10.1016/s0014-5793(02)02882-x), respectively. Annotations were performed with Snap Gene.
(PDF)

S6 Fig Survival assays in the presence of Naps. (A) Synthetic mature peptides (mNap) were tested against *N*. *cinerea* CCUG 346T. (B) Synthetic scrambled versions of the mature peptides (mNap_SCR_) were tested on *N*. *gonorrhoeae* FA1090. Bacteria were cultured in 96-well plates in protein-free spermidine-free GW medium for 3 h before peptides were added at various concentrations (arrow). At specific time points (0, 3, 6, 9, 24, 30, 36 h), one well per condition was emptied and serial diluted before plating on chocolate agar plates and incubation overnight. Colony-forming units (CFUs) were then counted. Dotted lines represent the limit of detection. (C) In order to check whether mNaps had a synergistic or competitive effect, they were tested against *N*. *gonorrhoeae* FA1090 with a fixed concentration of 2.5 μm each, alone or mixed. Data shown here represent the differences (in log scale) between t 0 and 9 h (exponential phase of growth) and t 9 and 36 h (autolytic phase) (*n* = 3). No synergistic or competitive effect was observed for any of the peptides. Note that the standard deviation at t 36 h was larger than usual due to recovery on GCB plates instead of chocolate agar (see Materials and methods). The data underlying this figure can be found in S9 Data.
(PDF)

S7 Fig Autolysis in 50 mM HEPES buffer (pH 8.5). (A) Against *N*. *gonorrhoeae* in plain buffer. The data shown here are the mean values of 8 biological replicates, except for Δ*napRABC napI*::*kanR* (*n* = 4). Standard deviation are shown in transparent corresponding colours. One-way ANOVA was performed on data from T = 75 min, with Dunnett’s multiple comparison against the WT values (*p* < 0.033, *; *p* < 0.002, **; *p* < 0.001, ***). (B) Against *N*. *gonorrhoeae* in buffer supplemented with 10 mM MgCl_2_ (*n* = 3). (C) Against *N*. *cinerea* in plain buffer (*n* = 3). (D) Against *N*. *cinerea* in buffer supplemented with 10 mM MgCl_2_ (*n* = 3). The data underlying this figure can be found in S10 Data.
(PDF)

S8 Fig Autolysis in buffer. Transmission electron microscopy images of samples prior to performing autolysis in buffer experiment (T = 0 min).
(PDF)

S9 Fig Cytotoxicity of Naps on THP-1 cells. LDH release assays were performed in the presence of 1 and 5 μm of each mNap with non-activated or activated THP-1 derived macrophages. No significant cytotoxic activity was detectable for any mNap (one-sample t and Wilcoxon test); error bars, SD of assays performed in triplicate. The data underlying this figure can be found in S11 Data.
(PDF)

S1 Table Predicted sequences of NapC and NapR. (XLSX)

S2 Table Nap genes in Neisseria spp. (XLSX)

S3 Table Strains and primers used in this study. (XLSX)

S1 Data Disc diffusion assays of antimicrobials and Naps against bacteria as indicated. (XLSX)

S2 Data Results of MICs in μg/ml. Strains are indicated.
(XLSX)

S3 Data Effect of mNaps on survival of wild-type *N*. *gonorrhoeae*. (XLSX)

S4 Data Effect of mNaps on survival of *N*. *gonorrhoeae*. Strains are indicated.
(XLSX)

S5 Data Effect of mNaps on lysis of RBCs. (XLSX)

S6 Data Growth curves in GCB medium. Strains and values of OD_600_ are indicated.
(XLSX)

S7 Data Results of co-culture of prey bacteria with *N*. *gonorrhoeae*. Strains are indicated, and survival is shown as CFU/ml.
(XLSX)

S8 Data Gene expression of the *nap* island in *N*. *gonorrhoeae*. RT-qPCR of expression of genes (indicated) in wild-type *N*. *gonorrhoeae* FA1090.
(XLSX)

S9 Data Survival assays in the presence of individual and combinations of Naps. Survival is shown as CFU/ml, with the folders corresponding to the panels in S6 Fig.
(XLSX)

S10 Data Autolysis in 50 mM HEPES buffer. Results show the OD540 of bacteria in buffers as indicated. The folders correspond to the panels in S7 Fig.
(XLSX)

S11 Data Cytotoxicity of Naps on THP-1 cells. Results of LDH release (measured by the OD_490_ of assays on cell supernatants) from THP-1 cells in the presence of individual mNaps. Lysis buffer shows the LDH release from 100% of cells.
(XLSX)

## Abbreviations

CE: Correia element
CFU: colony-forming unit
HGT: horizontal gene transfer
IHF: integration host factor
LB: lysogeny broth
LDH: lactate dehydrogenase
MBC: minimal bactericidal concentration
MDM: monocyte-derived macrophage
Naps: *Neisseria* autolysis peptides
OM: outer membrane
PCD: programmed cell death
RBC: red blood cell
TEM: transmission electron microscopy
