# Supplementary material for: Bacteriocin-like peptides encoded by a horizontally acquired island mediate Neisseria gonorrhoeae autolysis
Source: PLoS Biol. 2025 Feb 5;23(2):e3003001. doi: 10.1371/journal.pbio.3003001 (PMC11798529; doi:10.1371/journal.pbio.3003001)
Supplement: S1 Fig — DNA and amino acid sequences are given for each nap genes, as well as predicted alpha-fold structures and extra information, such as C39 peptidase cleaving sites, phase variable sequences based on available genomic data (details available in S1 Table) or gene alignments. Note that the alignment between napI and the Lactococcin-G immunity protein lagC was performed after the serendipitous observation that NapP was annotated as homolog to the “Lactococcin-G-processing and transport ATP-binding protein” LagD from Lactococcus lactis both in the genome of N. gonorrhoeae FA19 (GenBank accession no. CP012026.1, locus tag: VT05_00181) and N. gonorrhoeae 35/02 (GenBank accession no. CP012028.1, locus tag: WX61_01768). (PDF) [file pbio.3003001.s001.pdf]

Suppl. Fig 1

NapF

DNA sequence (in *Ng* FA1090, NGO_0432):

Atgaaacgtatctttttgcccgccttgcccgccatcctgcctttatccgcttatgccgacctgcccttgacgattgaagacataatgaccgacaagggaaaatggaaactggaaacttcccttacctatctgaatagcgaaaacagccgcgccgcacttgccgcaccggtttacattcaaaccggcgcaacctcgtttatccccattccgaccgaaattcaagaaaacggcagcaataccgatatgctcgccggcacgctcggtttgcgctacggactgaccggcaataccgacatttacggcagcggcagctatctgtggcacgaagaacgcaaactcgacggcaacggcaaaacccgcaacaaacggatgtccgacatatccgccggcatcagccacaccttccttaaagacggcaaaaaccccgccctaatcagctttcttgaaagcacggtttacgaaaaatcgcgcaacaaagcctcgttaatcaaaaaaaggggggctttgccccttttataacttaaggataaattatgaatattaa

Amino acid sequence:

MKRIFLPALPAILPLSAYADLPLTIEDIMTDKGKWKLETSLTYLNSENSRAALAAPVYIQTGATSFIPIPTEIQENGSNTDMLAGTLGLRYGLTGNTDIYGSGSYLWHEERKLDGNGKTRNKRMSDISAGISHTFLKDGKNPALISFLESTVYEKSRNKASLIKKRGLCPFYNLRINYEY

Predicted alpha-fold structure (homodimer, as predicted by ColabFold (Mirdita *et al*, 2022)) :

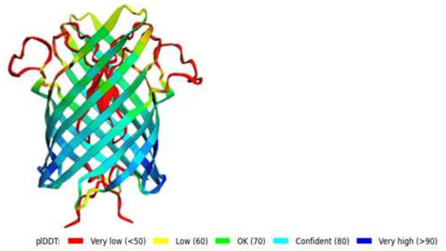

Other information: N-term signal peptide (1-19) not shown on predicted structure.

NapR

DNA sequence (in *Ng* FA1090, NGO_02290):

Atgggggggcaatatagttctttagaaaattttgctttatccgtactaagggaaaaaattataaacaatactgtttccagtgatgaaaaattattgattattaatgatttttttatcaggttttcttgagtttgatcctgagacaggggaacctgtcggggagacgcttaaaattgagcaaatgattgatttcttttttagtctaa

Amino acid sequence:

MGGQYSSLENFALSVLREKIINNTVSSDEKLLIINDFLSGFLEFDPETGEPVGETLKIEQMIDFFLV

Predicted alpha-fold structure (as displayed on UniProt, 04/01/2023):

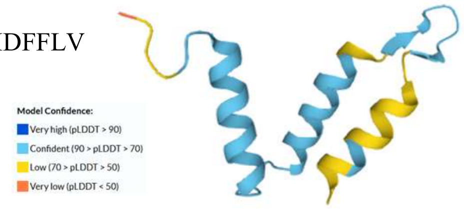

Other information:

On Uniprot (04/01/2023), predicted to be a COesterase domain-containing protein or a site-specific DNA-methyltransferase (adenine-specific) by automatic annotation.; on Phyre2 (04/01/2023), low homology (39.4% confidence, 41% ID) with a DNA binding protein (c4dt1B template).

Clustal Omega Alignment based on pblast result (https://blast.ncbi.nlm.nih.gov/Blast.cgi) : note that the 2 versions of NapR correspond to phase variable-dependent alleles.

```
CDS93995.1-conserved_hypothetical_protein-[Clostridioides_difficile]    MNIKNEIVALISKKIDGGQYSSLENFALSVLREKIIKNTVSSDEKLLIINDFLSGFLEFD  60
NapR_Ng-FA1090-NGO_02290                                                ---------------MGGQYSSLENFALSVLREKIINNTVSSDEKLLIINDFLSGFLEFD  45
NapR_Ng-MS11-NGFG_RS02295                                               MNIKNEIVALISKKIDGGQYSSLENFALSVLREKIINNTVSSDEKLLIINDFLSGFLEFD  60
                                                                                        ********************:***********************

CDS93995.1-conserved_hypothetical_protein-[Clostridioides_difficile]    PETGKPVGETLKIEQMIDFFLV        82
NapR_Ng-FA1090-NGO_02290                                                PETGEPVGETLKIEQMIDFFLV        67
NapR_Ng-MS11-NGFG_RS02295                                               PETGEPVGETLKIEQMIDFFLV        82
                                                                        ****:*****************
```

Phase variability (⁑, C39 peptidase predicted cleavage site)

atgaatattaaaaatgaaatagttgctttaatttctaaaaaaattgat[g]$_n$caatatagttctttagaaaattttgctttatccgtactaagggaaaaaattataaacaatactgtttccagtgatgaaaaattattgattattaatgatttttttatcaggttttcttgagtttgatcctgagacaggggaacctgtcggggagacgcttaaaattgagcaaatgattgatttcttttttagtctaa

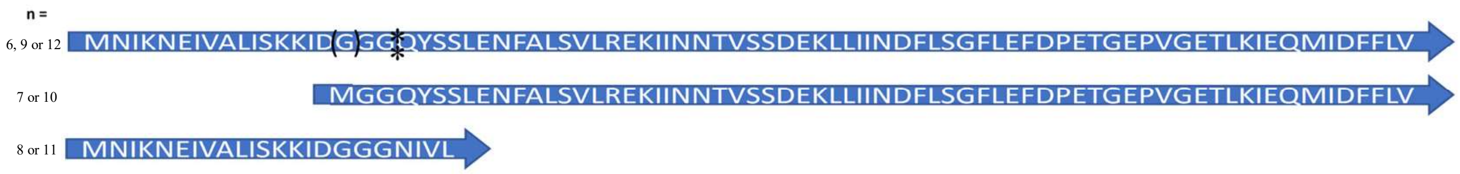

Poly-G tract	Phenotype	Number of isolates out of total (%)
7 or 10	Mature NapR (leaderless)	6548/10854 (60.3%)
6, 9 or 12	Complete NapR (with leader sequence)	3179/10854 (29.3%)
8 or 11	Leader sequence only	525/10854 (4.8%)
Fixed	Complete NapR (GG**A**GGG)	6/10854 (0.06%)
Undetermined	Unknown	59610854 (5.5%)

NapA

DNA sequence (in *Ng* FA1090, NGO_0430):

Ttggatgatctaatactatatttttttatcaggtatattcggaaatcaagttgctgaatatattataaaaaacaaccgtgagataaaagttccctttattgcgctttacgctatattttttacgttgatttatactgtggctttgt tgtttctcagtctgatctattgggtcaatggtgcggagattgcatggaaggggataggtattttcagtatgtcggtcagtttttgtatagtcttctgtctttatttgattgacaaggcaggaagatgtaaggataagaaa caatag

Amino acid sequence (⁑, C39 peptidase predicted cleavage site, in green, important residues for leader peptide based on Aucher *et al*, 2005 and Havarstein *et al,* 1994) :

MDDLILYFLSGIFGNQVAEYIIKNNREIKVPFIALYAIFFTLIYTVALLFLSLIYWVNGA⁑EIAWKGIGIFSMSVSFCIVFCLYLIDKAG RCKDKKQ

Predicted alpha-fold (as displayed on UniProt, 04/01/2023):

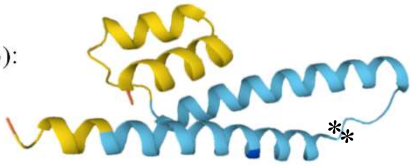

NapB

DNA sequence (in *Ng* FA1090, not annotated, NEIS3237):

ttgggtctaggctcaacctgcgtttgctgcatctttggaagaaatagtggattcgaaaaaataggaagaattctgattaatggtatgcgaggtgtagcagtgggggctgttgctggtggcataactggttatgcag gttctacgggtaagaacactgacattcgccgttaa

Amino acid sequence (⁑, C39 peptidase predicted cleavage site, in green, important residues for leader peptide):

MGLGSTCVCCIFGRNSGFEKIGRILINGMRGVAVGAVAGG⁑ITGYAGSTGKNTDIRR

Predicted alpha-fold (as predicted by ColabFold (Mirdita *et al*, 2022)): 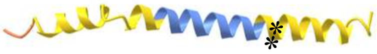

NapC

DNA sequence (in *Ng* FA1090, NGO_02280):

gtgtttgctgttattttcttttcgacattgggctgtattttagcatggataagagatattcctaaaataaaatcaaaaaaaaattttggcaagatccttatatattataggaataataaatgtaataatcagctatgtattaatt aaaaatatattggtttctgtttcggatggaggtggaataaaatatgttgcaatatatttatcaaatcttttttttggacagtattgatgtatgttcttgtgaaaagattatctaaaaagccaagctga

Amino acid sequence (⁑, C39 peptidase predicted cleavage site, in green, important residues for leader peptide) :

MFAVIFFSTLGCILAWIRDIPKIKSKKILARSLYIIGIINVIISYVLIKNILVSVSDGG⁑GIKYVAIYLSNLFFWTVLMYVLVKRLSKKPS

Predicted alpha-fold (as displayed on UniProt, 04/01/2023):

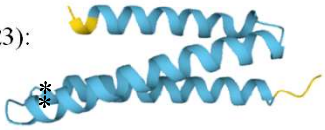

Other information:

Phase variability

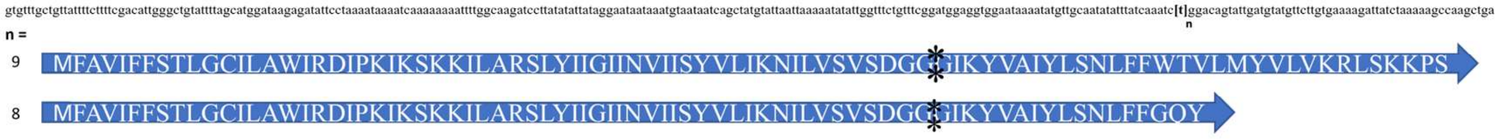

Poly-T tract	Phenotype	Number of isolates out of total (%)
9	Cationic NapC	819/10854 (7,6%)
8	Non cationic NapC	9318/10854 (85,9%)
7, 10 or early stop codon	Non cationic NapC	11/10854 (0.11%)
Undetermined	Unknown	706/10854 (6.5%)

Clustal Alignment by MUSCLE based on psi-blast result (https://blast.ncbi.nlm.nih.gov/Blast.cgi) : note that the 2 versions of NapC correspond to phase variable-dependent alleles and that the viral protein sequence can be retrieved with GenBank accession number DAS81996.1.

```
DAS81996_Inoviridae        MFPVIFFSTLGCILVWIKDMPKIKSKKILGISLYIIGIINVIIGYVLIKNILVSVSDEIG
cationic_NapC_NgFA1090     MFAVIFFSTLGCILAWIRDIPKIKSKKILARSLYIIGIINVIISYVLIKNILVSVSDGGG
NONcationic_NapC_MS11      MFAVIFFSTLGCILAWIRDIPKIKSKKILARSLYIIGIINVIIGYVLIKNILVSVSDGGG
                           **.***********.**.*:*********.  ************.*************   *

DAS81996_Inoviridae        IKYLAIYLSNIFFGQS--------------
cationic_NapC_NgFA1090     IKYVAIYLSNLFFWTVLMYVLVKRLSKKPS
NONcationic_NapC_MS11      IKYVAIYLSNLFFGQY--------------
                           ***:******:**
```

NapI
DNA sequence (in *Ng* FA1090, NGO_0429):
Ttggaattgaaatactgcaatcgggaggctaaggcttccgttgtaacgtttaccatatatttcattattttaatttcatcaaaaaacaaaattcaagattatacataccgccaatcgtttcttgattgttttgtatag

Amino acid sequence:
MELKYCNREAKASVVTFTIYFIILISSKNKIQDYTYRQSFLDCFV

Predicted alpha-fold structure (as displayed on UniProt, 04/01/2023): : 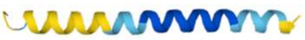

```
LagC_immunity_lactococcinG_GeneBan-FJ938036.1      MFNNIVVFINFLSFVFILVGVDIKYNDNRIKIVHVTFFISFILVMLTSLISHNSIAYSLS 60
NapI_putative-immunity-NGO_0429                    --------------------MELKYCNREAKASVVTFTIYFIILISSK-NKIQDYTYRQS 39
                                                                       :::** :.. *   *** * **::: :.  . :. :*  *

LagC_immunity_lactococcinG_GeneBan-FJ938036.1      QILEILCIICILLLFYILKKTNSLSNRANVVFIIFIVTQVIIIINQLFIR   110
NapI_putative-immunity-NGO_0429                    ---FLDCFV-----------------------------------------   45
                                                      : *::
```

NapH
DNA sequence (in *Ng* FA1090, NGO_0428):
atgatgaagaacaaatatattttaataaaagcagcaatcggcataaccgccatatccatattttatcttctgactcaaggaagcatcggcaaaactgaagaaccatcatatttcttgatgttcatgtttctcaactcgc tttggtttgaagaaaataaaacagttatggctgccgttacggcaatgattgccgcccactttatatttgtcgcactatccgattaa

Amino acid sequence :
MMKNKYILIKAAIGITAISIFYLLTQGSIGKTEEPSYFLMFMFLNSLWFEENKTVMAAVTAMIAAHFIFVALSD

Predicted alpha-fold structure (as displayed on UniProt, 04/01/2023): 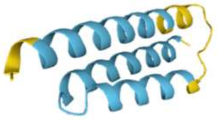

NapP
DNA sequence (in *Ng* FA1090, NGO_0427):
atggaacaaaaaaggcggtttgccgcttccctgctcctcgccgccgccctgccgctttgtgcacattcttttccctttgcggaagaaaaccccatagcctacggcaaagtcaaaatacaaagctggaaagcgc ggcgggatttcaatattgtaaagcaggatttggatttttcctgcggggcggcttcggtggcgacgcttttgaacaatttttacgggcaaacgctgacggaagaagaagtgttggaaaaactgggtaaggaaca gatgcgcgcgtcgtttgaggatatgcggcgcattatgcccgatttgggttttgaggcgaagggctatgccctgtctttcgagcagctcgcgcagttgaaaatccccgtcatcgtgtatctgaaataccgcaagg atgatcatttctcggtattgcgcggcataggcggcaatacggttttgcttgccgacccgtcgccgggtcatgtttcgatgagcagagcgcagtttttggaggcttggcaaacccgtgagggaaatttggcaggc aaaattttggcggtcgtgccgaaaaaagcggaggcgatttcaaataaattgtttttcacacatcatcccaagcggcagacggagtttgcagtcggacaaatcaggcaagggcgcgcagagtaa

Amino acid sequence:
MEQKRRFAASLLLAAALPLCAHSFPFAEENPIAYGKVKIQSWKARRDFNIVKQDLDFSCGAASVATLLNNFYGQTLTEEEVLEKL GKEQMRASFEDMRRIMPDLGFEAKGYALSFEQLAQLKIPVIVYLKYRKDDHFSVLRGIGGNTVLLADPSPGHVSMSRAQFLEAWQ TREGNLAGKILAVVPKKAEAISNKLFFTHHPKRQTEFAVGQIRQGRAE

Predicted alpha-fold structure (as displayed on UniProt, 04/01/2023):

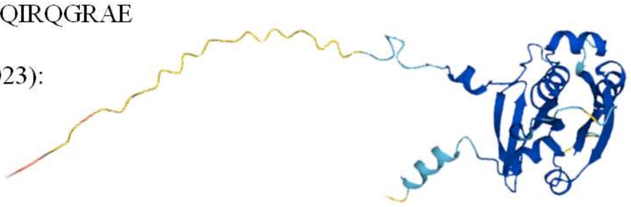

Other information: N-term signal peptide (1-23).

Suppl. Fig 1. Supplementary information regarding genes of the Nap island in *Neisseria gonorrhoeae* FA1090. DNA and amino acid sequences are given for each *nap* genes, as well as predicted alpha-fold structures and extra information, such as C39 peptidase cleaving sites, phase variable sequences based on available genomic data (details available in Suppl. Table 1) or gene alignments. Note that the alignment between *napI* and the Lactococcin-G immunity protein *lag*C was performed after the serendipitous observation that NapP was annotated as homolog to the "Lactococcin-G-processing and transport ATP-binding protein" LagD from *Lactococcus lactis* both in the genome of *N. gonorrhoeae* FA19 (GenBank accession no. CP012026.1, locus tag: VT05_00181) and *N. gonorrhoeae* 35/02 (GenBank accession no. CP012028.1, locus tag: WX61_01768).
