# Supplementary material for: Bacteriocin-like peptides encoded by a horizontally acquired island mediate Neisseria gonorrhoeae autolysis
Source: PLoS Biol. 2025 Feb 5;23(2):e3003001. doi: 10.1371/journal.pbio.3003001 (PMC11798529; doi:10.1371/journal.pbio.3003001)
Supplement: S2 Fig — In both Nm and Nl strains, NapF sequences correspond to the long NEIS0907 allele, which is found in another loci in N. gonorrhoeae (Ng) (under the gene name NGO_0166 in Ng FA1090). In Nm, chromosomal rearrangements led to the fusion between the 2 NEIS0907-containing loci. Genes in purple correspond to those homologous to the NGO_0166-containing locus in Ng FA1090. In Nl, the presence of the long NEIS0907 allele correlates with the loss of napA. Regarding napB, due to the presence of an earlier start codon, it is present as a longer allelic form than in Nm or Ng. As for napI, it is slightly shorter in Nl than in Ng. Note that an extra gene is represented in black in the locus of Nl (NLA_13780, function unknown); a homolog sequence exists in Ng FA1090 but the start codon is not present. As shown in Fig 1C, the locus is absent in Nc. Instead, 3 genes of unknown function (light grey) are present between the flanking NEIS0794 and NEIS0808. ⁑, C39 peptidase predicted cleavage site. Note that for NapB in Nl, ⁑ was not positioned as alternative GG-sites might be at play. Sequences were manually annotated on Snap Gene viewer. (PDF) [file pbio.3003001.s002.pdf]

Suppl. Fig 2

Nm C58

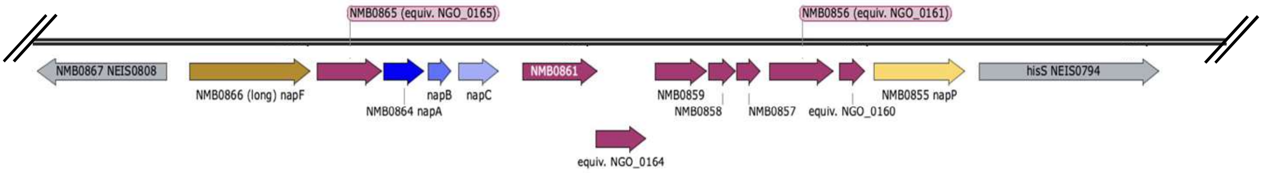

NapF:
MKRIFLPALPAILPLSTYADLPLTIEDIMTDKGKWKLETSLTYLNSENNRAELAAPVYIQTGATSFIPIPTEIQENGSNTDMLVGTLGLRYGLTG
NTDIYGSGSYLWHEERKLDGNSKTRNKRMSDVSLGISHTFLKDDKNPALISFLESTVYEKSRNKASSGKSWLIGATTYKAIDPIVLSLTAAYRIN
GSKTLSDGIRYKSGNYLLLNPNISFAANDRISLTGGIQWLGRQPDRTDGKRESSRNTSTYAHFGAGFGFTKTTALNASARFNVSGQSSSELKF
GVQHTF
NapA :
MDDLILYFLSGIFGNQVAEYIIKNNREIKVPFIVLYAIFFTLIYTVALLFLSLIYWVNGA⁑EIAWKGIGIFSMSVSFCIVFCLYLIDKAGRCKDKKQ
NapB:
MGLGSTCVCCIFGRNSGFEKIGRILINGMRGVAVGTVAGG⁑INGYAGSTGKNTDIRR
NapC (polyT-tract of 9):
MEVRGGKVVFAVIFFSTLGCILAWIRDIPKIKSKKILARSLYIIGIINVIISYVLIKNILVSVSDGG⁑GIKYVAIYLSNLFFWTVLMYVLVKRLSKKPS
NapP:
MDNMMKFKYVFLLACVVVSLSYRLNAAPMFNDNPVVYGKIKVQSWKARRDFNIVKQDLDFSCGAASVATLLNNFYGQTLTEEEVLKKLD
KEQMRASFEDMRRIMPDLGFEAKGYALSFEQLAQLKIPVIVYLKYRKDDHFSVLRGIDGNTVLLADPSLGHVSMSRAQFLDAWQTREGNL
AGKILAVIPKKAETISNKLFFTQHPKRQTEFTVGQIRQARAE

Nm FAM18

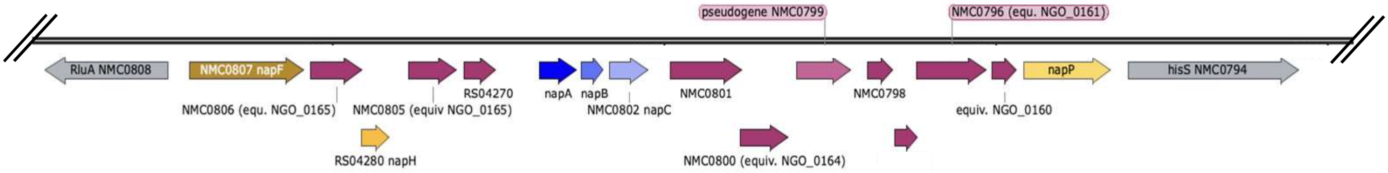

NapF:
MKRIFLSALPAILPLSTYADLPLTIEDIMTDKGKWKLETSLTYLNSENSRAALAAPVYIQTGATSFIPIPTEIQENGSNTDMLAGTLGLRYGLTGN
TDIYGSGSYLWHEERKLDGNGKTRNKRMSDVSLGISHTFLKDDKNPALISFLESTVYEKSRNKASSGKSWLIGATTYKAIDPIVLSLTAAYRING
SKTLSDGIRYKSGNYLLLNPNISFAANDRISLTGGIQWLGKQPDRIDGKKESARNTSTYAHFGAGFGFTKTTALNASARFNVSGQSSSELKLG
VQHTF
NapH:
MVNKYIKTAISITAISIFYLLTQESSGKTEEPSYFLMFMFLNSLWFEENKTVMAAVTAMIAAHLIFVTLSD
NapA :
MDDLILYFLSGIFGNQVAEYIIKNNREIKVPFIVLYAIFFTLIYTVALLFLSLIYWVNGA⁑EIAWKGIGIFSMSVSFCIVFCLYLIDKAGRCKDKKQ
NapB:
MGLGSTCVCCIFGRNSGFEKIGRILINGMRGVAVGTVAGG⁑ITGYAGSTGKNTDIRR
NapC (polyT-tract of 9):
MEVRGGKVVFAVIFFSTLGCILAWIRDIPKIKSKKILARSLYIIGIINVIISYVLIKNILVSVSDGG⁑GIKYVAIYLSNLFFWTVLMYVLVKRLSKKPS
NapP:
MDNMMKFKYVFLLACVVVSLSYRLNAAPMFNDNPVVYGKIKVQSWKERRDFNIVKQDLDFSCGAASVATLLNNFYGQTLTEEEVLKKLDK
EQMRASFEDMRRIMPDLGFEAKGYALSFEQLAQLKIPVIVYLKYRKDDHFSVLRGIDGNTVLLADPSLGHVSMSRAQFLDAWQTREGNLA
GKILAVVPKKAETISNKLFFTHHPKRQTEFAVGQIRQARAE

Nl 020-06

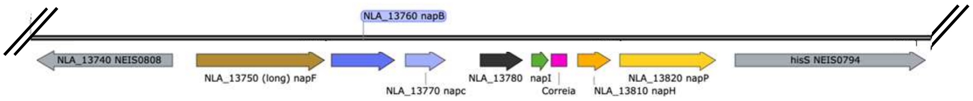

NapF:
MKRIFLSALPAILPLSAYADLPLTIEDIITDKGKWKLETSLTYLNSENSRAALAAPVYIQTGATSFIPIPTEIQENGSNTDMLAGTLGLRYGLTGNT
DIYGSGSYLWHEERKLDGNGKTRNKRMSDISAGISHTFLKDDKNPALIGFLESTVYEKSRNKASSGKSWLIGATTYKAIDPVVLSLTAAYRING
SKTLSSNTKYKAGNYWMLNPNISFAANDRISLTGGIQWLGKQPDRLDGKKESARNTSTYAHFGAGFGFTKTTALNASARFNVSGQSSSELK
FGVQHTF
NapB:
MKKQITAAVMMLSMITPAMANGLDNQAFEDQVFHTRADAPMQLAELSQKEMKETEGEWIPNAVGGIMGGVGGHFSYMASAIASGSY
NREAHWATIGGGALIGAMNPINGGRALINGMRGVAVGTVAGGITGYAGSTGKNTDIRR
NapC:
MEVRGGKVVFAVIFFSTLGCILAWIRDIPKIKSKKILARSLYIIGIINVIIGYVLIKNILVSVSDGG⁑GIKYVAIYLSNLFFWTVLMYVLVKRLSKKPS
NapI:
MELKYWNRKAKASIITFTIYFIILISSKNKIQDYTYRQ
NapH:
MHEKPYKMMKNKYILIKAAIGITAISIFYLLTQESSGKTEEPSYFLMFMFLNLLWFEENKTVMAAVTAMITTHLIFVALSD
NapP:
MEQKRRFAASLLLAAVLPLCAHSFPFAEENPIAYGKVKIQSWKERRDFNIVKQDLDFSCGAASVATLLNNFYGQTLTEEEVLKKLDKEQMRA
SFEDMRRIMPDLGFEAKGYALSFEQLAQLKIPVIVYLKYRKDDHFSVLRGVDGNTVLLADPSLGHVSMSRAQFLDAWQTREGNLAGKILAVI
PKKAETISNKLFFTQHPKRQTEFAVEQIRQARAE

Nc NCTC10294

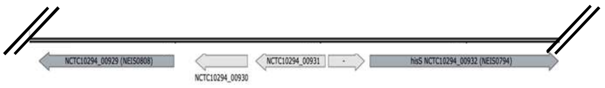

Suppl. Fig 2. Nap island organization in *Neisseria meningitidis (Nm)* MC58, *Nm* FAM18, *Neisseria lactamica (Nl)* 020-06 and *Neisseria cinerea* (*Nc*) NCTC10294. In both *Nm* and *Nl* strains, NapF sequences correspond to the long NEIS0907 allele, which is found in another loci in *N. gonorrhoeae (Ng)* (under the gene name NGO_0166 in *Ng* FA1090). In *Nm*, chromosomal rearrangements led to the fusion between the two NEIS0907-containing loci. Genes in purple correspond to those homologous to the NGO_0166-containing locus in *Ng* FA1090. In *Nl,* the presence of the long NEIS0907 allele correlates with the loss of *napA*. Regarding *napB*, due to the presence of an earlier start codon, it is present as a longer allelic form than in *Nm* or *Ng*. As for *napI*, it is slightly shorter in *Nl* than in *Ng*. Note that an extra gene is represented in black in the locus of *Nl* (NLA_13780, function unknown) ; a homolog sequence exists in *Ng* FA1090 but the start codon is not present. As shown in Fig 1C, the locus is absent in *Nc*. Instead, 3 genes of unknown function (light grey) are present between the flanking NEIS0794 and NEIS0808. ⁑, C39 peptidase predicted cleavage site. Note that for NapB in *Nl*, ⁑ was not positioned as alternative GG-sites might be at play. Sequences were manually annotated on Snap Gene viewer.
