# Supplementary material for: Bacteriocin-like peptides encoded by a horizontally acquired island mediate Neisseria gonorrhoeae autolysis
Source: PLoS Biol. 2025 Feb 5;23(2):e3003001. doi: 10.1371/journal.pbio.3003001 (PMC11798529; doi:10.1371/journal.pbio.3003001)
Supplement: S3 Fig — (A) Growth curves in GCB medium. Markerless deletion strains (plain lines, ML) are compared to resistant marker strains (dotted lines, K for kanamycin resistance marker and E for erythromycin resistance marker). Standard deviation are shown in lighter colours (n = 9). The data underlying this figure can be found in S6 Data. (B) Schematic representation of the classical method to generate deletion mutants in N. gonorrhoeae. Briefly, an overlap PCR product is generated from upstream region (PCR 1), downstream region (PCR 2), and resistance cassette marker (PCR 3) amplifications. The Overlap PCR product is then used for natural transformation into N. gonorrhoeae and the mutant strain is selected based on the acquired antibiotic resistance. (C) Schematic representation of the markerless method. Briefly, 2 overlap PCR products are generated from upstream region (PCR 1) and downstream region (PCR 2) as well as, for the first product only, an endogenous promoter (popaB) followed by section/counter-selection markers (PCR 3) amplifications. Note that the pheS* sequence used here was amplified from the genome of N. gonorrhoeae FA1090 itself and point mutations (* = T275S and A318G) were then introduced by overlap PCR. The markerless method consists in first selecting a recombinant colony through kanamycin resistance selection, and secondly, by transforming this colony with markerless overlap PCR product to allow the counter-selection of the pheS* marker. (PDF) [file pbio.3003001.s003.pdf]

Suppl. Fig 3

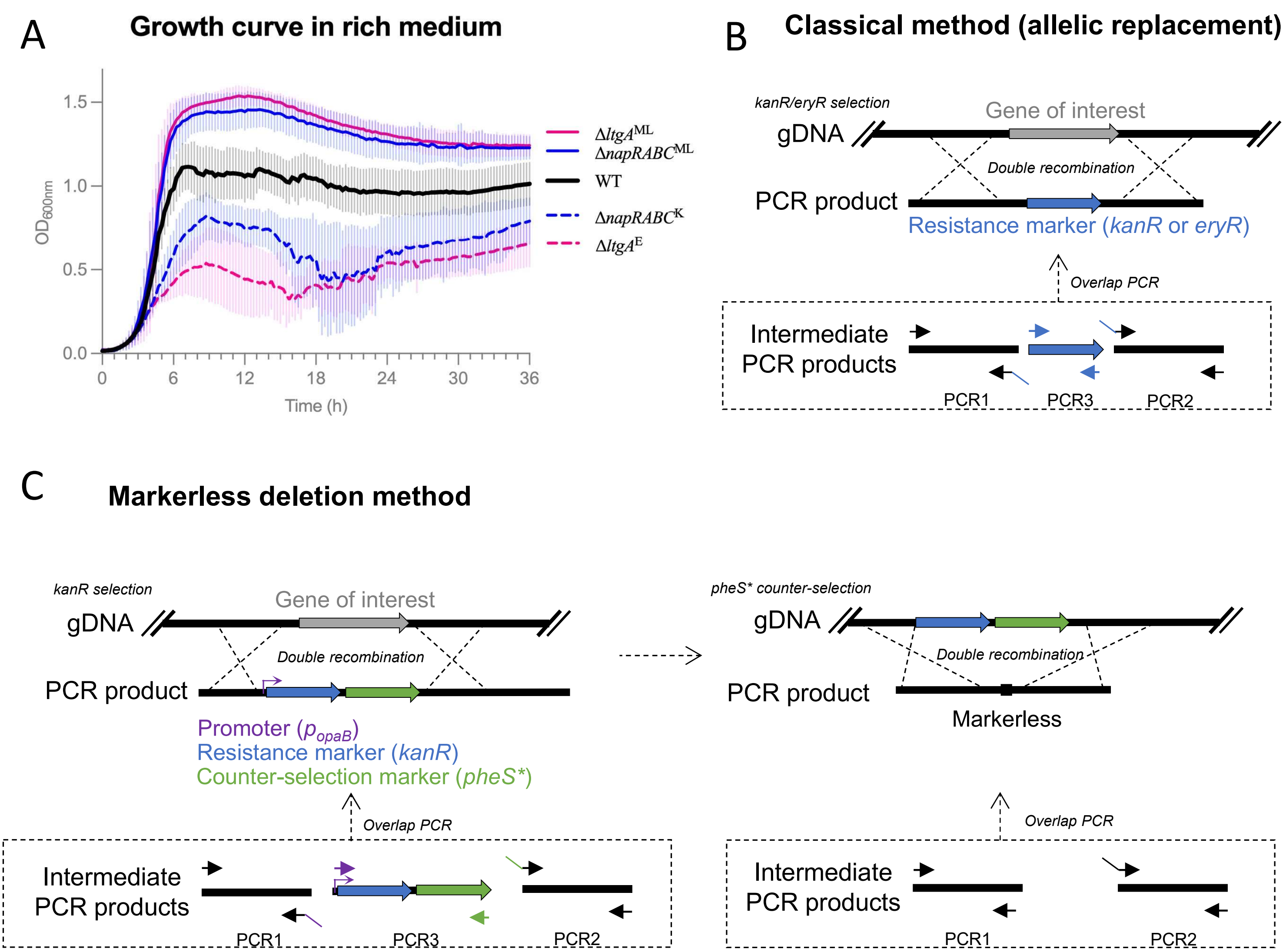

Suppl. Fig 3. Construction and characterization of deletion mutants in *N. gonorrhoeae*. A. Growth curves in GCB medium. Markerless deletion strains (plain lines, ^{ML}) are compared to resistant marker strains (dotted lines, ^{K} for kanamycin resistance marker and ^{E} for erythromycin resistance marker). Standard deviation are shown in lighter colours (n = 9). **B.** Schematic representation of the classical method to generate deletion mutants in *N. gonorrhoeae*. Briefly, an overlap PCR product is generated from upstream region (PCR 1), downstream region (PCR 2) and resistance cassette marker (PCR 3) amplifications. The Overlap PCR product is then used for natural transformation into *N. gonorrhoeae* and the mutant strain is selected based on the acquired antibiotic resistance. **C.** Schematic representation of the markerless method. Briefly, two overlap PCR products are generated from upstream region (PCR 1) and downstream region (PCR 2) as well as, for the first product only, an endogenous promoter (p_{opaB}) followed by section/counter-selection markers (PCR 3) amplifications. Note that the *pheS** sequence used here was amplified from the genome of *N. gonorrhoeae* FA1090 itself and point mutations (* = T275S and A318G) were then introduced by overlap PCR. The markerless method consists in first selecting a recombinant colony through kanamycin resistance selection, and secondly, by transforming this colony with markerless overlap PCR product to allow the counter-selection of the *pheS** marker.
