# Supplementary material for: Bacteriocin-like peptides encoded by a horizontally acquired island mediate Neisseria gonorrhoeae autolysis
Source: PLoS Biol. 2025 Feb 5;23(2):e3003001. doi: 10.1371/journal.pbio.3003001 (PMC11798529; doi:10.1371/journal.pbio.3003001)
Supplement: S4 Fig — Prey bacteria were incubated alongside either wild-type N. gonorrhoeae (WT FA1090, black bars) or the ΔnapRABC strain (blue bars) for 3 or 24 h in FB medium at a 1:1 ratio. Prey bacteria were then recovered selectively on agar plates and CFU/ml were counted. Data were normalised against the recovery of prey bacteria grown without the gonococcus (100%). The data underlying this figure can be found in S7 Data. Multiple paired t test were performed between each pair (WT vs. ΔnapRABC) with no significant difference in their survival (n = 3, error bars, SD). (PDF) [file pbio.3003001.s004.pdf]

Suppl. Fig 4

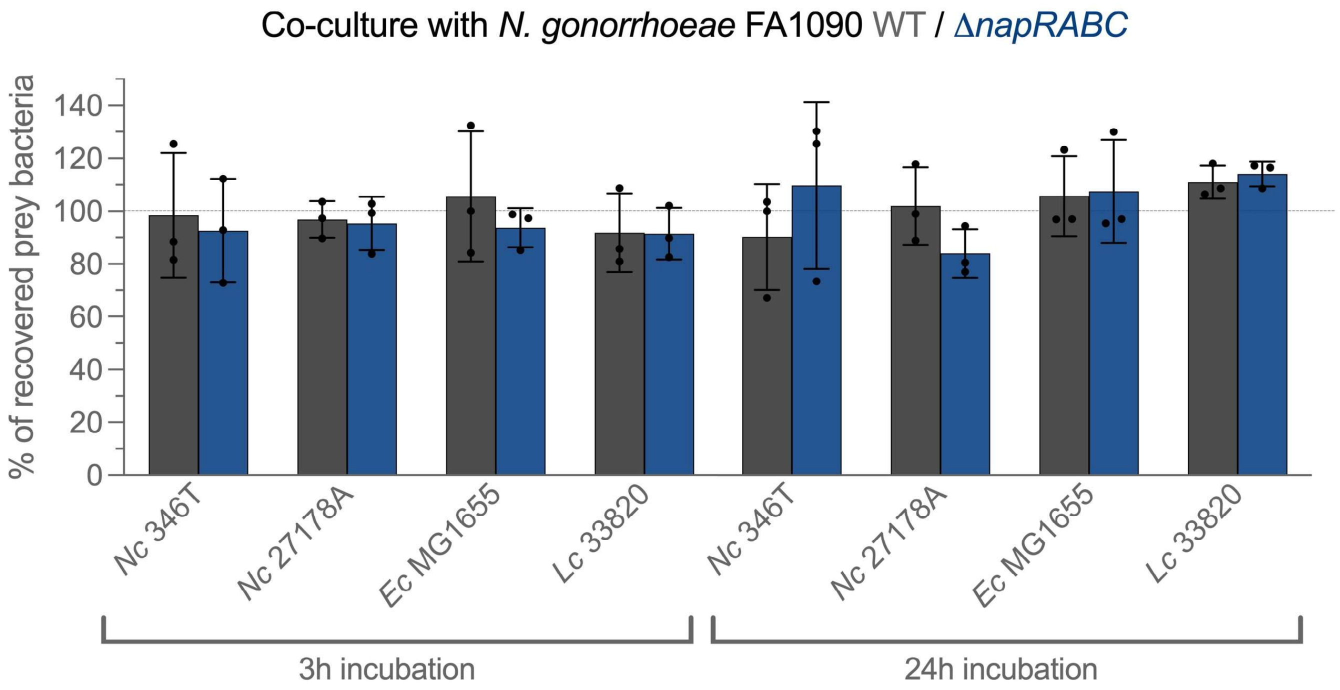

Suppl. Fig 4. Co-culture of prey bacteria with *N. gonorrhoeae*. Prey bacteria were incubated alongside either wild-type *N. gonorrhoeae* (WT FA1090, black bars) or the Δ*napRABC* strain (blue bars) for 3 or 24 hours in FB medium at a 1:1 ratio. Prey bacteria were then recovered selectively on agar plates and CFU/mL were counted. Data were normalised against the recovery of prey bacteria grown without the gonococcus (100%). Multiple paired *t* test were performed between each pair (WT *vs.* Δ*napRABC*) with no significant difference in their survival (n = 3, error bars, SD).
