# Supplementary material for: Bacteriocin-like peptides encoded by a horizontally acquired island mediate Neisseria gonorrhoeae autolysis
Source: PLoS Biol. 2025 Feb 5;23(2):e3003001. doi: 10.1371/journal.pbio.3003001 (PMC11798529; doi:10.1371/journal.pbio.3003001)
Supplement: S5 Fig — (A) RT-qPCR on all genes of the nap locus of strain FA1090. Samples were recovered from GW liquid cultures in flasks at different time points, and gene expression was normalised based on samples recovered from plates and resuspended in GW (T0). Error bars represent standard deviation from the mean (n = 3–4). One-way ANOVA was performed on data with minimum 2-fold change (dotted lines) compared to T0 (p < 0.033, *; p < 0.002, **; p < 0.001, ***). Growth curves were performed in parallel (right bottom panel). The data underlying this figure can be found in S8 Data. (B) Predicted ribosome binding sites (RBS) and promoter regions (grey arrows) in the nap locus of strain FA1090. RBS were manually annotated based on the presence of a GGA enriched sequence −3 to −10 of an ATG start codon; promoter regions predicted by BPROM (http://softberry.com). (C, D) Pink arrows represent repeats of Correia elements (CE), while blue boxes show integration host factor (IHF) binding sites, as defined previously (10.1016/s0378-1119(01)00725-9 and 10.1016/s0014-5793(02)02882-x), respectively. Annotations were performed with Snap Gene. (PDF) [file pbio.3003001.s005.pdf]

Suppl. Fig 5

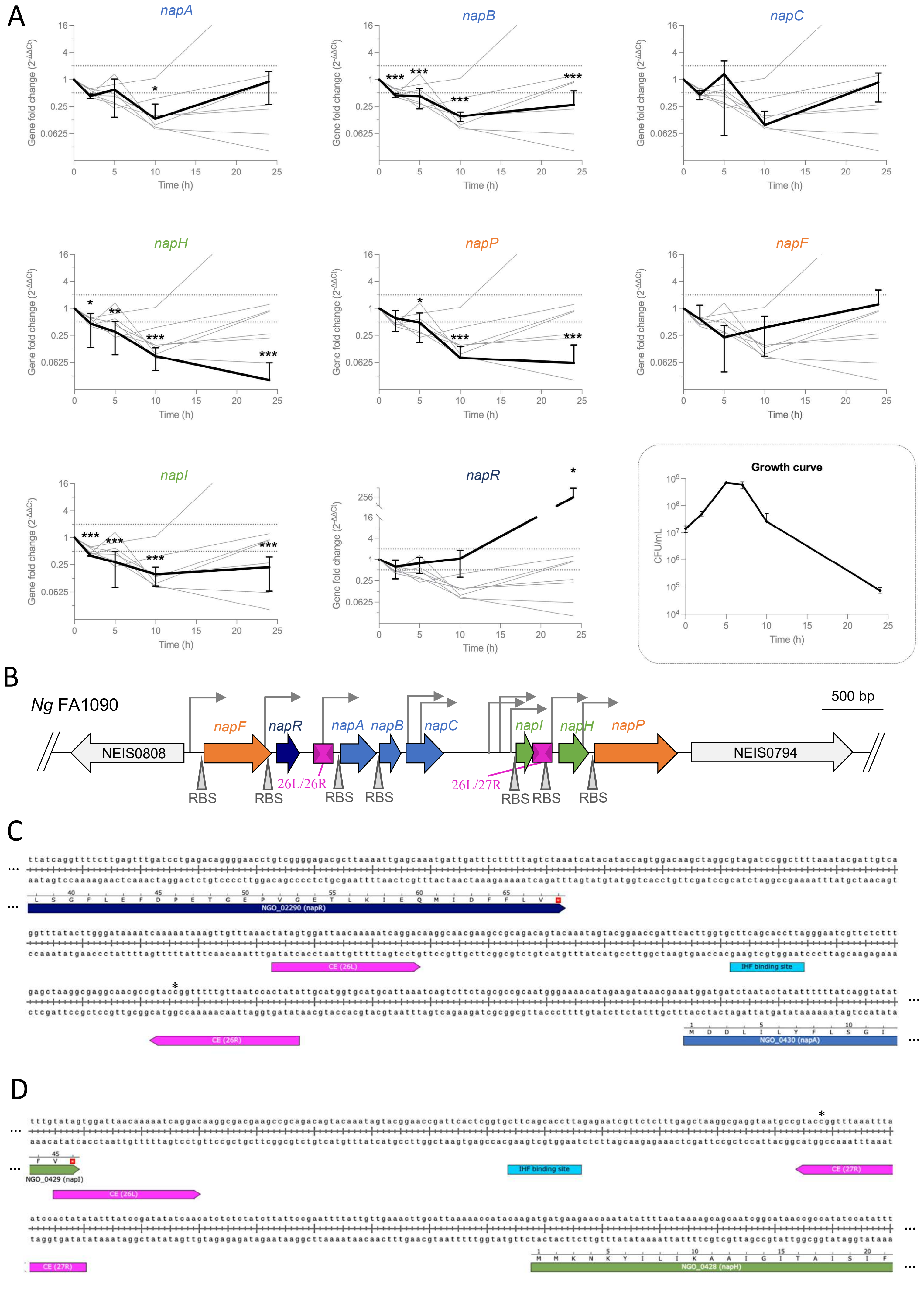

A
napA
napB
napC
napH
napP
napF
napI
napR
Gene fold change (2-ΔΔCt)
Time (h)
Growth curve
CFU/mL
B
Ng FA1090
500 bp
NEIS0808
napF
napR
napA
napB
napC
napI
napH
napP
NEIS0794
26L/26R
26L/27R
RBS
C
NGO_02290 (napR)
CE (26L)
IHF binding site
CE (26R)
NGO_0430 (napA)
D
NGO_0429 (napI)
CE (26L)
IHF binding site
CE (27R)
NGO_0428 (napH)

Suppl. Fig 5. Gene expression of the *nap* island in *N. gonorrhoeae*. A. RT-qPCR on all genes of the *nap* locus of strain FA1090. Samples were recovered from GW liquid cultures in flasks at different time points, and gene expression was normalized based on samples recovered from plates and resuspended in GW (T0). Error bars represent standard deviation from the mean (n = 3-4). One-way ANOVA was performed on data with minimum 2-fold change (doted lines) compared to T0 ($p < 0.033$, *; $p < 0.002$, **; $p < 0.001$, ***). Growth curves were performed in parallel (right bottom panel). **B.** Predicted ribosome binding sites (RBS) and promoter regions (grey arrows) in the *nap* locus of strain FA1090. RBS were manually annotated based on the presence of a GGA enriched sequence -3 to -10 of an ATG start codon; promoter regions predicted by BPROM (http://softberry.com). **C.** and **D**. Pink arrows represent repeats of Correia elements (CE), while blue boxes show integration host factor (IHF) binding sites, as defined previously (10.1016/s0378-1119(01)00725-9 and 10.1016/s0014-5793(02)02882-x), respectively. Annotations were performed with Snap Gene.
