# Supplementary material for: Bacteriocin-like peptides encoded by a horizontally acquired island mediate Neisseria gonorrhoeae autolysis
Source: PLoS Biol. 2025 Feb 5;23(2):e3003001. doi: 10.1371/journal.pbio.3003001 (PMC11798529; doi:10.1371/journal.pbio.3003001)
Supplement: S6 Fig — (A) Synthetic mature peptides (mNap) were tested against N. cinerea CCUG 346T. (B) Synthetic scrambled versions of the mature peptides (mNapSCR) were tested on N. gonorrhoeae FA1090. Bacteria were cultured in 96-well plates in protein-free spermidine-free GW medium for 3 h before peptides were added at various concentrations (arrow). At specific time points (0, 3, 6, 9, 24, 30, 36 h), one well per condition was emptied and serial diluted before plating on chocolate agar plates and incubation overnight. Colony-forming units (CFUs) were then counted. Dotted lines represent the limit of detection. (C) In order to check whether mNaps had a synergistic or competitive effect, they were tested against N. gonorrhoeae FA1090 with a fixed concentration of 2.5 μm each, alone or mixed. Data shown here represent the differences (in log scale) between t 0 and 9 h (exponential phase of growth) and t 9 and 36 h (autolytic phase) (n = 3). No synergistic or competitive effect was observed for any of the peptides. Note that the standard deviation at t 36 h was larger than usual due to recovery on GCB plates instead of chocolate agar (see Materials and methods). The data underlying this figure can be found in S9 Data. (PDF) [file pbio.3003001.s006.pdf]

Suppl. Fig 6

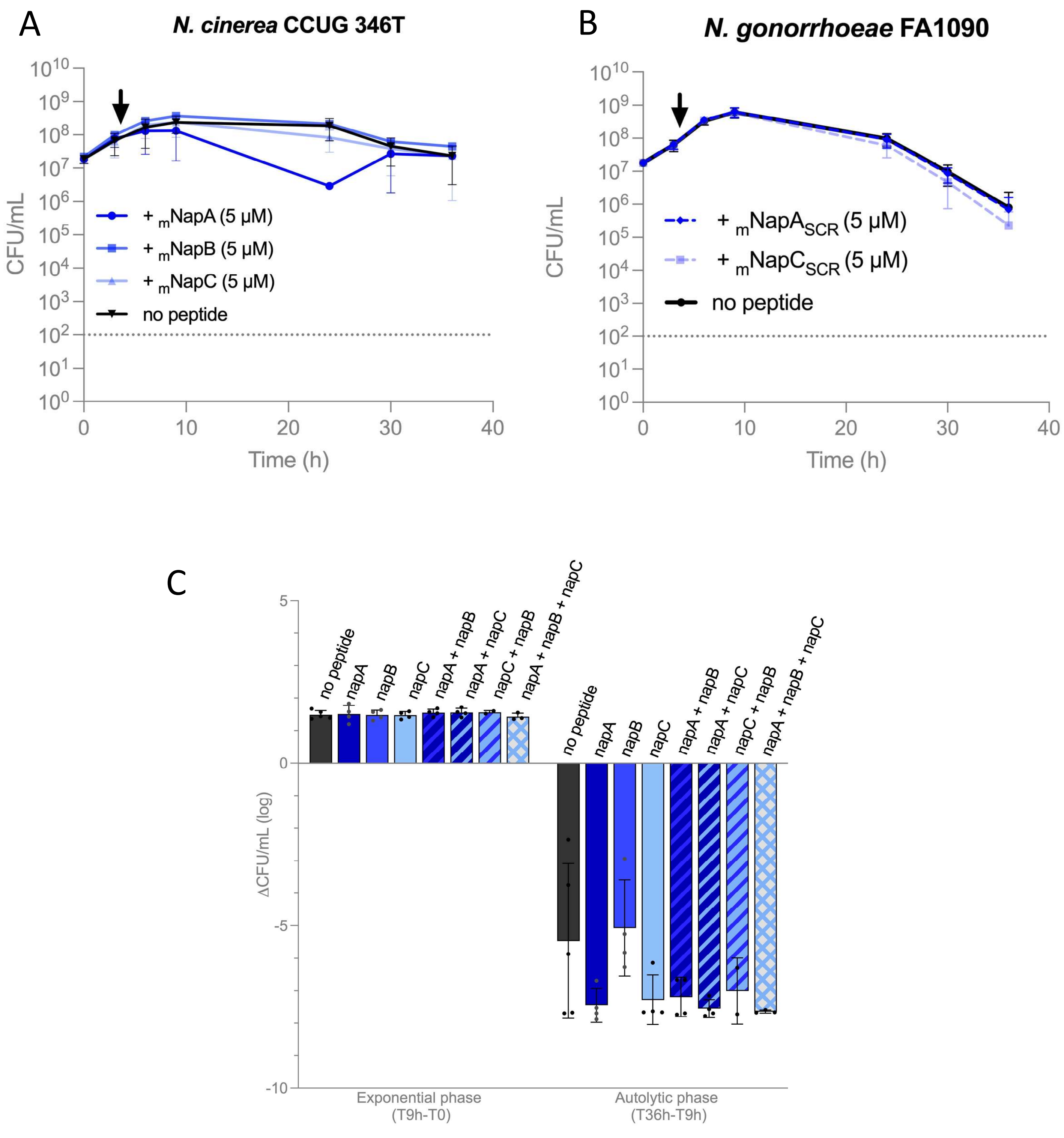

Suppl. Fig 6. Survival assays. A. Synthetic mature peptides (mNap) were tested against *N. cinerea* CCUG 346T. **B.** Synthetic scrambled versions of the mature peptides ($mNap_{SCR}$) were tested on *N. gonorrhoeae* FA1090. Bacteria were cultured in 96 well-plates in protein-free spermidine-free GW medium for three hours before peptides were added at various concentrations (arrow). At specific time points (0, 3, 6, 9, 24, 30, 36 h), one well per condition was emptied and serial diluted before plating on chocolate agar plates and incubation overnight. Colony forming units (CFU) were then counted. Dotted lines represent the limit of detection. **C.** In order to check whether mNaps had a synergistic or competitive effect, they were tested against *N. gonorrhoeae* FA1090 with a fixed concentration of 2.5 µM each, alone or mixed. Data shown here represent the differences (in log scale) between t 0 and 9h (exponential phase of growth) and t 9 and 36h (autolytic phase) (n = 3). No synergistic or competitive effect was observed for any of the peptides. Note that the standard deviation at t 36h were bigger than usual due to recovery on GCB plates instead of chocolate agar (see material and method).
