# Supplementary material for: Bacteriocin-like peptides encoded by a horizontally acquired island mediate Neisseria gonorrhoeae autolysis
Source: PLoS Biol. 2025 Feb 5;23(2):e3003001. doi: 10.1371/journal.pbio.3003001 (PMC11798529; doi:10.1371/journal.pbio.3003001)
Supplement: S7 Fig — (A) Against N. gonorrhoeae in plain buffer. The data shown here are the mean values of 8 biological replicates, except for ΔnapRABC napI::kanR (n = 4). Standard deviation are shown in transparent corresponding colours. One-way ANOVA was performed on data from T = 75 min, with Dunnett’s multiple comparison against the WT values (p < 0.033, *; p < 0.002, **; p < 0.001, ***). (B) Against N. gonorrhoeae in buffer supplemented with 10 mM MgCl2 (n = 3). (C) Against N. cinerea in plain buffer (n = 3). (D) Against N. cinerea in buffer supplemented with 10 mM MgCl2 (n = 3). The data underlying this figure can be found in S10 Data. (PDF) [file pbio.3003001.s007.pdf]

**Suppl. Fig 7**

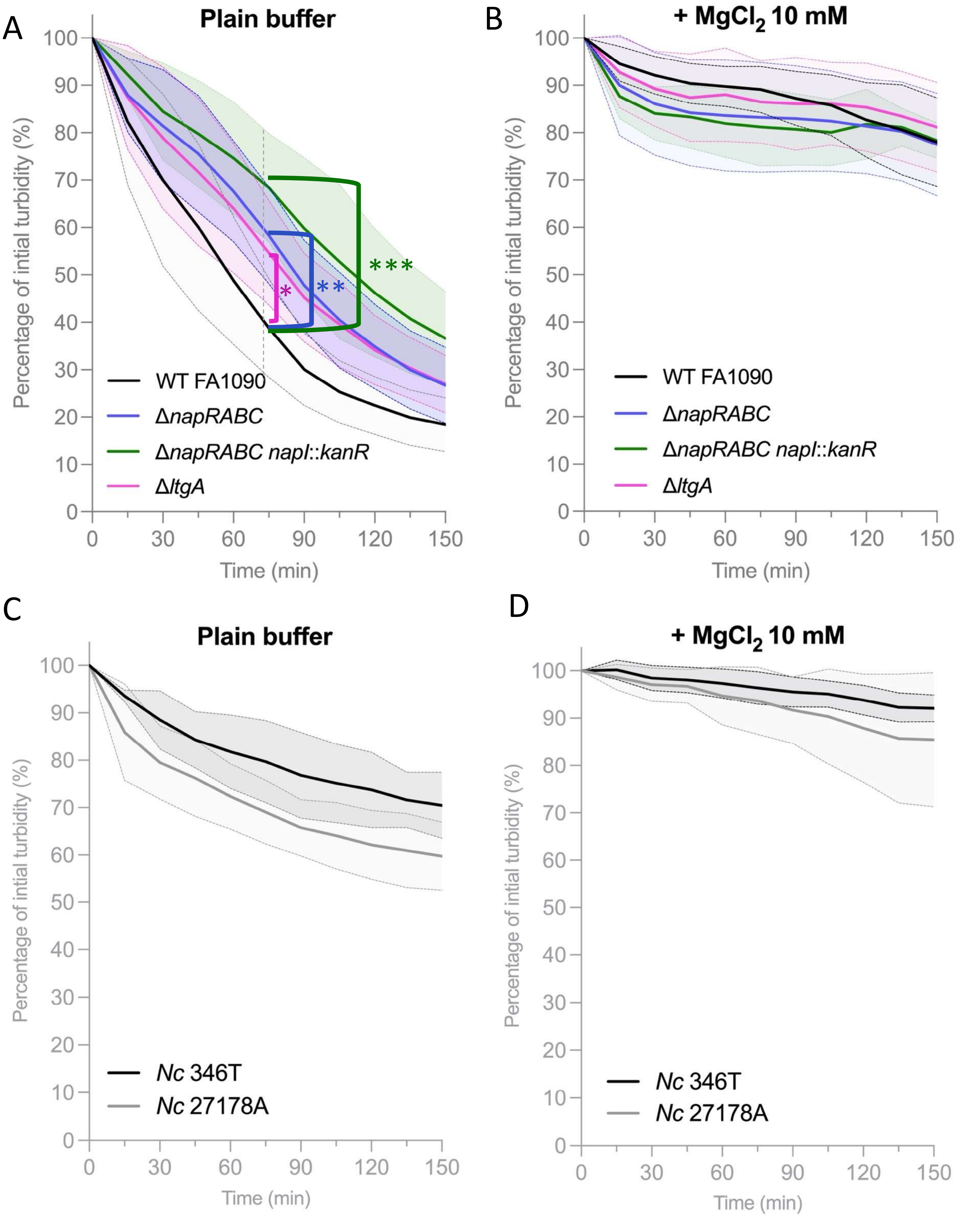


**Suppl. Fig 7. Autolysis in 50 mM HEPES buffer (pH 8.5). A.** Against *N. gonorrhoeae* in plain buffer. The data shown here are the mean values of 8 biological replicates, except for Δ*napRABC napI::kanR* (n = 4). Standartd deviation are shown in transparent corresponding colors. One-way ANOVA was performed on data from T = 75 min, with Dunnett's multiple comparison against the WT values ($p < 0.033$, *; $p < 0.002$, **; $p < 0.001$, ***). **B.** Against *N. gonorrhoeae* in buffer supplemented with 10 mM $MgCl_2$ (n = 3). **C.** Against *N. cinerea* in plain buffer (n = 3). **D.** Against *N. cinerea* in buffer supplemented with 10 mM $MgCl_2$ (n = 3).
