# Supplementary material for: Bacteriocin-like peptides encoded by a horizontally acquired island mediate Neisseria gonorrhoeae autolysis
Source: PLoS Biol. 2025 Feb 5;23(2):e3003001. doi: 10.1371/journal.pbio.3003001 (PMC11798529; doi:10.1371/journal.pbio.3003001)
Supplement: S8 Fig — Transmission electron microscopy images of samples prior to performing autolysis in buffer experiment (T = 0 min). (PDF) [file pbio.3003001.s008.pdf]

Suppl. Fig 8

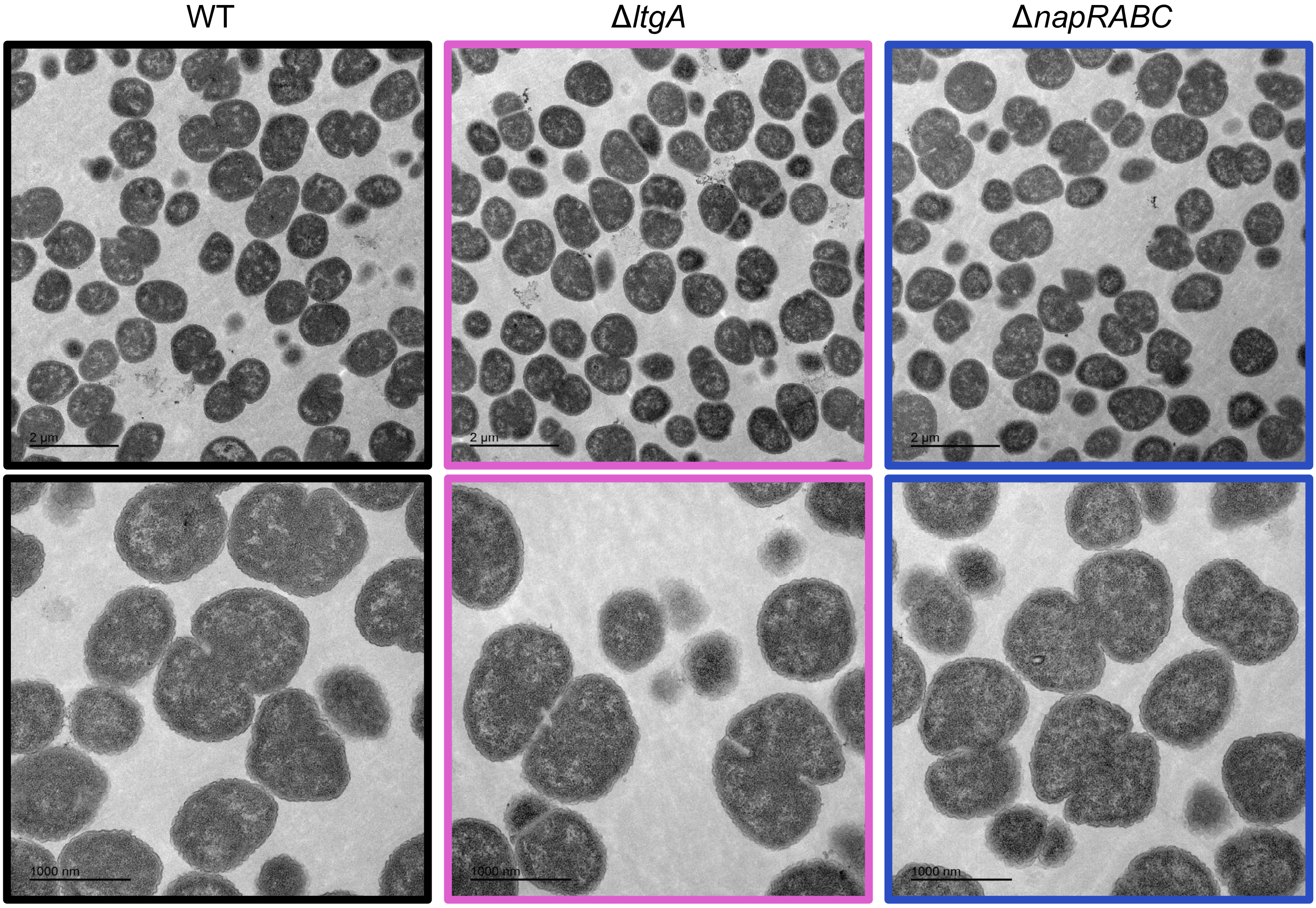

Suppl. Fig 8. Autolysis in buffer. Transmission electron microscopy images of samples prior to performing autolysis in buffer experiment (T = 0 min).
