# Supplementary material for: Bacteriocin-like peptides encoded by a horizontally acquired island mediate Neisseria gonorrhoeae autolysis
Source: PLoS Biol. 2025 Feb 5;23(2):e3003001. doi: 10.1371/journal.pbio.3003001 (PMC11798529; doi:10.1371/journal.pbio.3003001)
Supplement: S9 Fig — LDH release assays were performed in the presence of 1 and 5 μm of each mNap with non-activated or activated THP-1 derived macrophages. No significant cytotoxic activity was detectable for any mNap (one-sample t and Wilcoxon test); error bars, SD of assays performed in triplicate. The data underlying this figure can be found in S11 Data. (PDF) [file pbio.3003001.s009.pdf]

Suppl. Fig 9

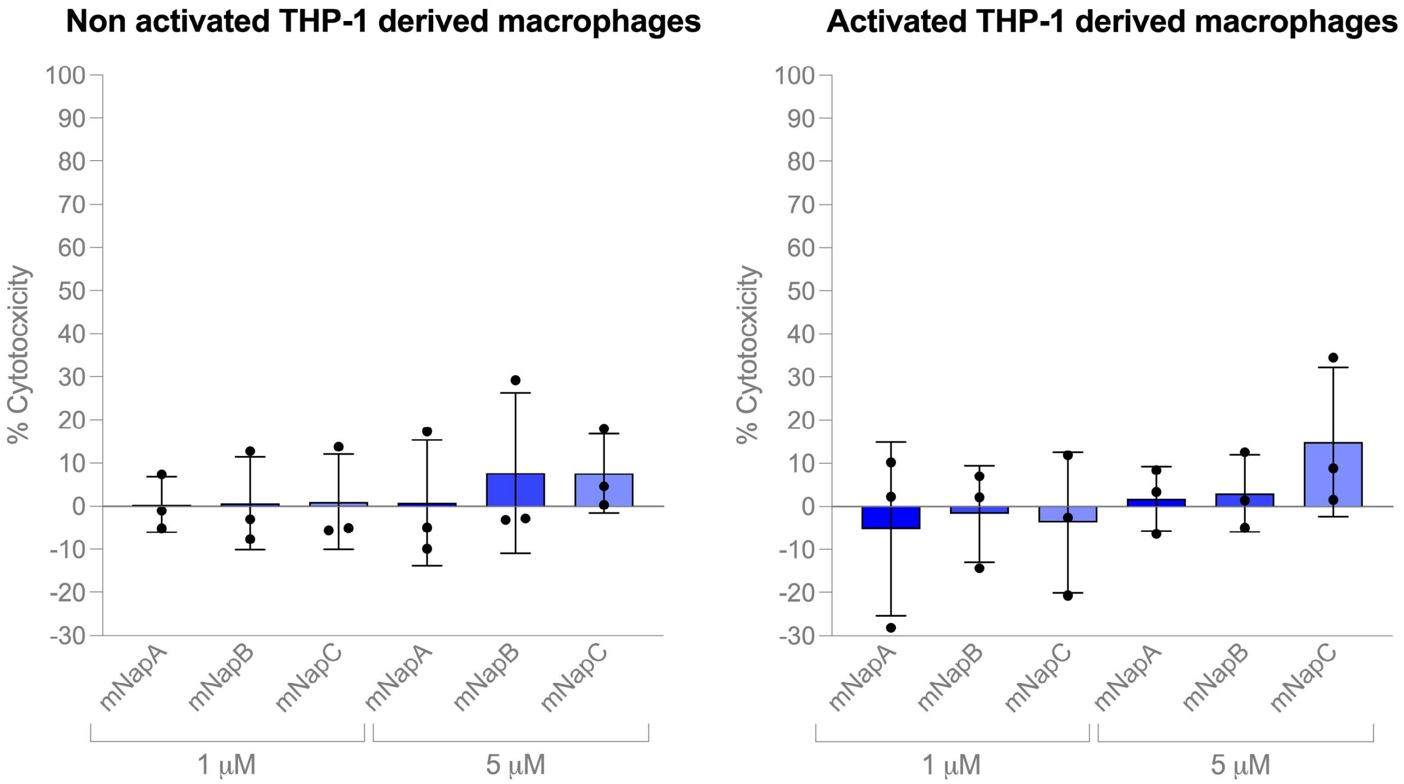

Suppl. Fig 9. Cytotoxicity of Naps on THP-1 cells. LDH release assays were performed in the presence of 1 and 5 µM of each mNap with non-activated or activated THP-1 derived macrophages. No significant cytotoxic activity was detectable for any mNap (One sample t and Wilcoxon test); error bars, SD of assays performed in triplicate.
